# Supplementary figures and images for: Chemotherapy resistance due to epithelial-to-mesenchymal transition is caused by abnormal lipid metabolic balance
Source: eLife. 2026 Jan 12;13:RP104374. doi: 10.7554/eLife.104374 (PMC12795503; doi:10.7554/eLife.104374)

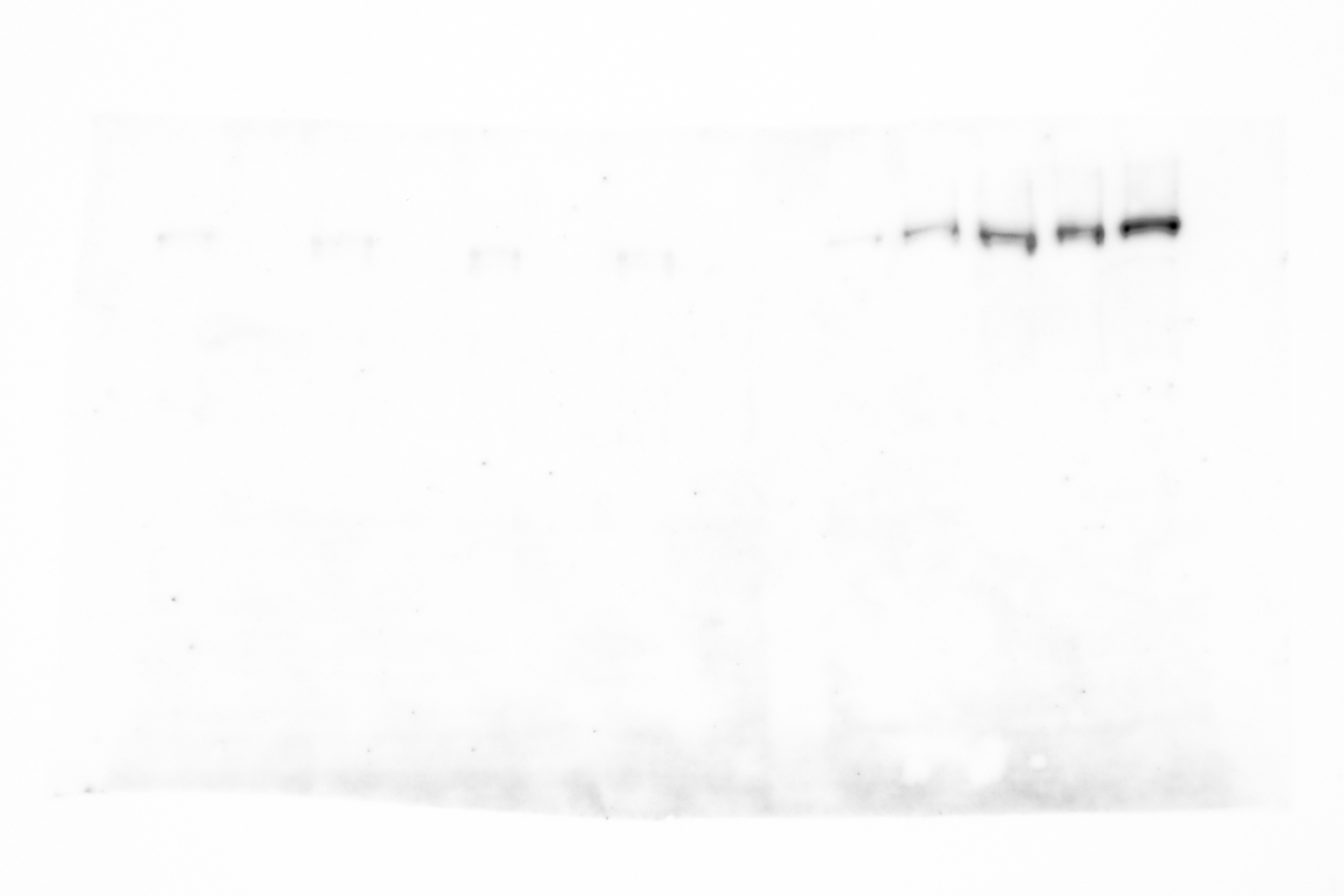

Supplement: Figure 1—source data 1. [file elife-104374-fig1-data1.zip › Figure1-source data 1/Figure 1B ABCA1.tif]

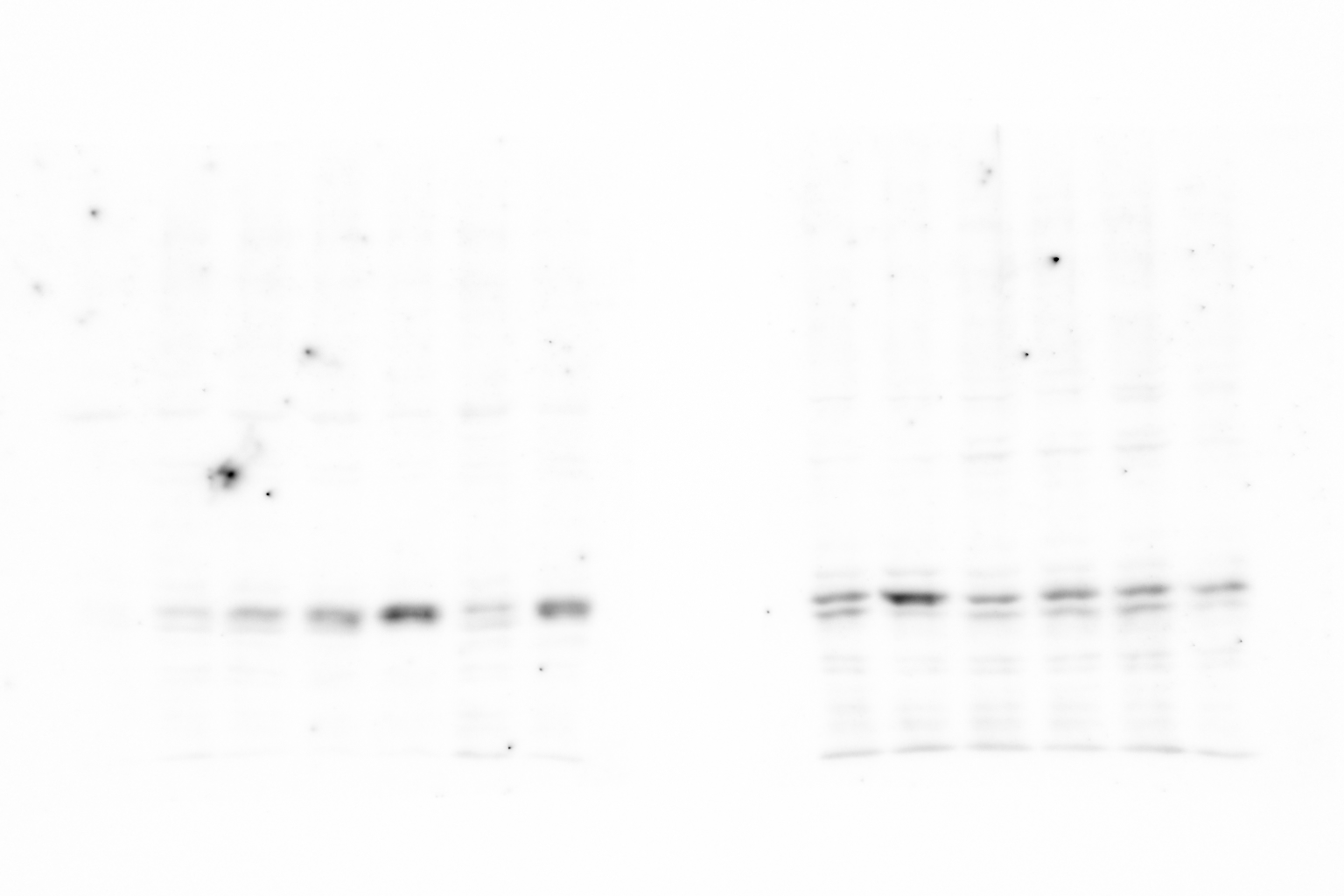

Supplement: Figure 1—source data 1. [file elife-104374-fig1-data1.zip › Figure1-source data 1/Figure 1B Snail.tif]

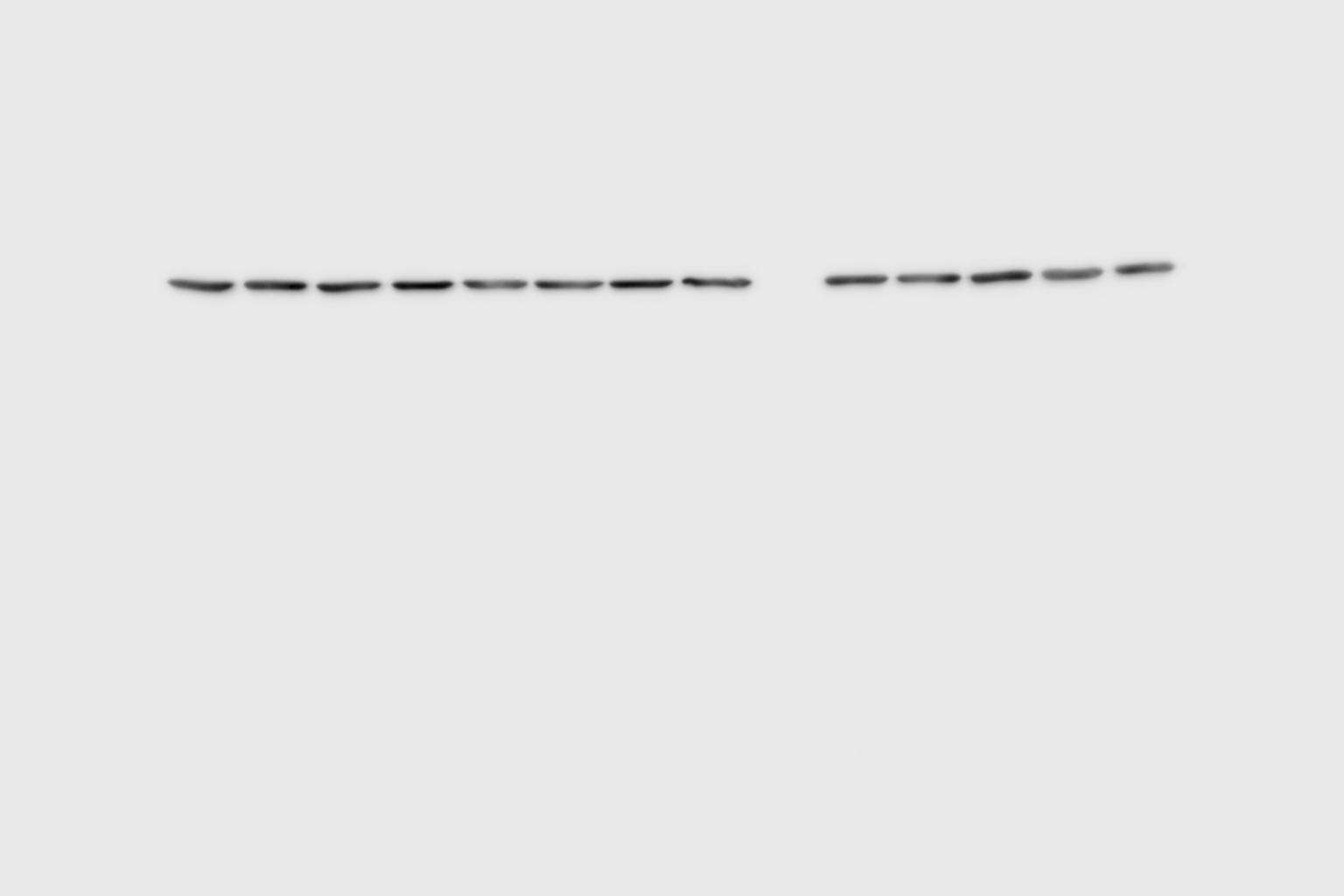

Supplement: Figure 1—source data 1. [file elife-104374-fig1-data1.zip › Figure1-source data 1/Figure 1B tubulin.tif]

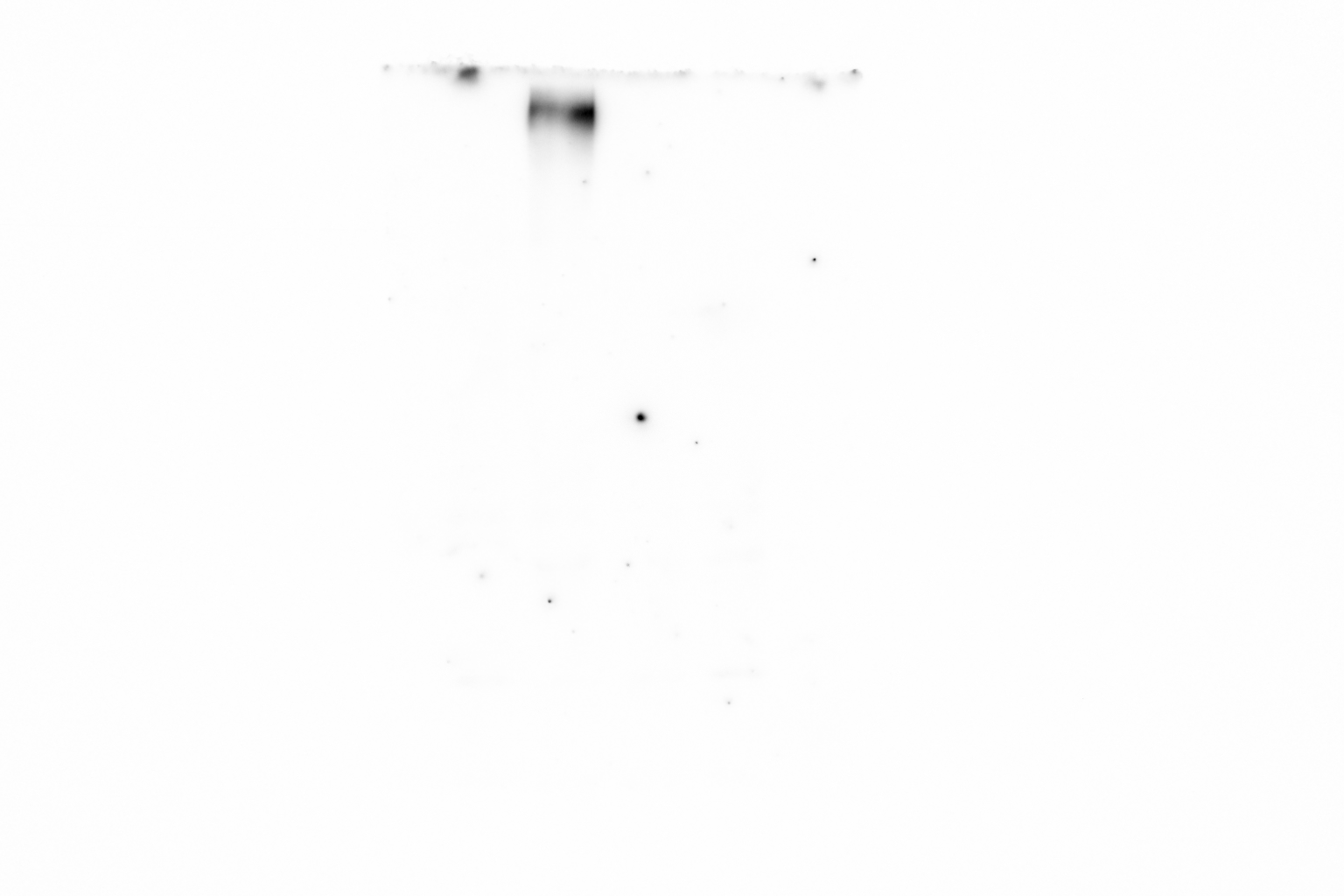

Supplement: Figure 1—source data 1. [file elife-104374-fig1-data1.zip › Figure1-source data 1/Figure 1L ABCA1.tif]

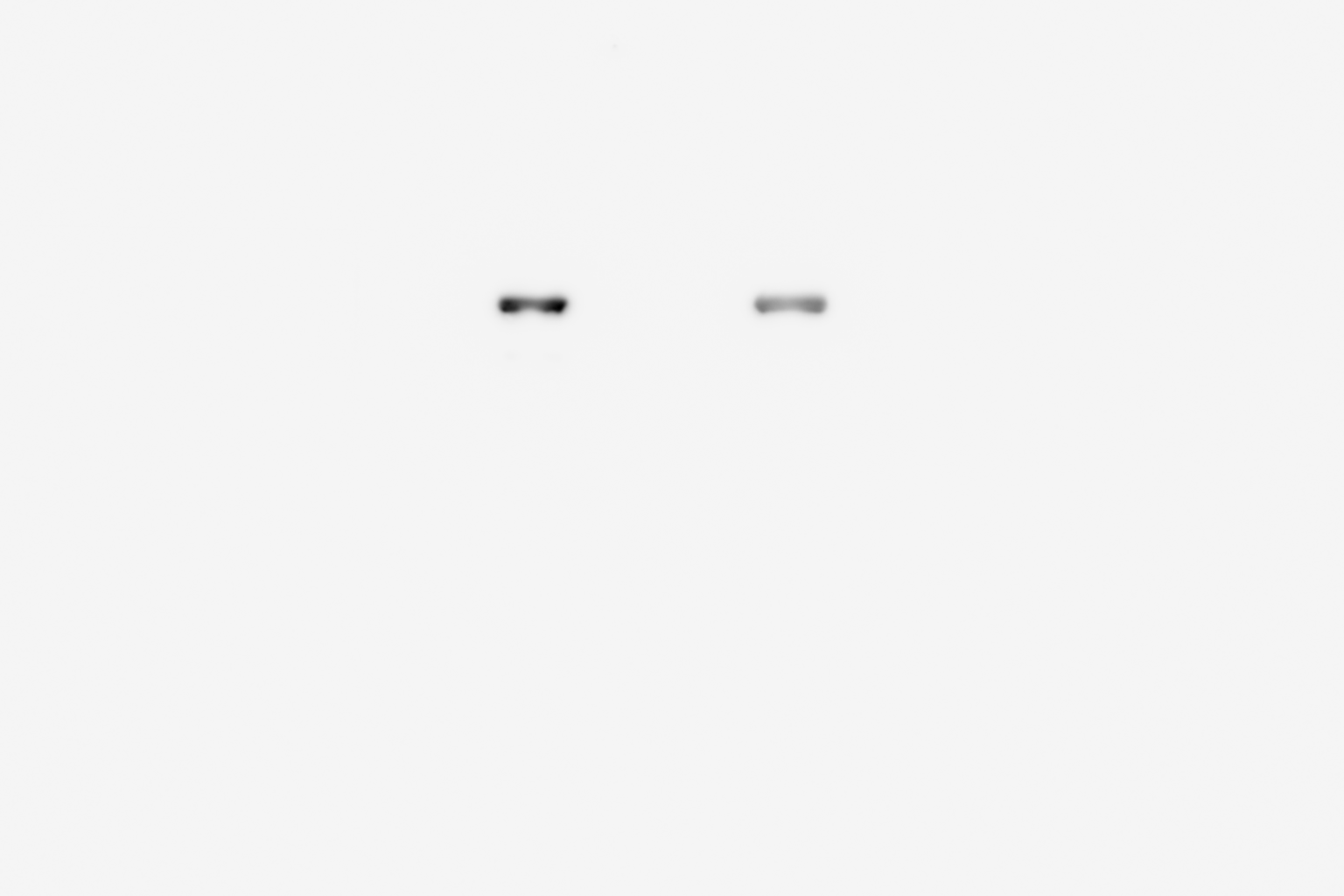

Supplement: Figure 1—source data 1. [file elife-104374-fig1-data1.zip › Figure1-source data 1/Figure 1L E-cadherin.tif]

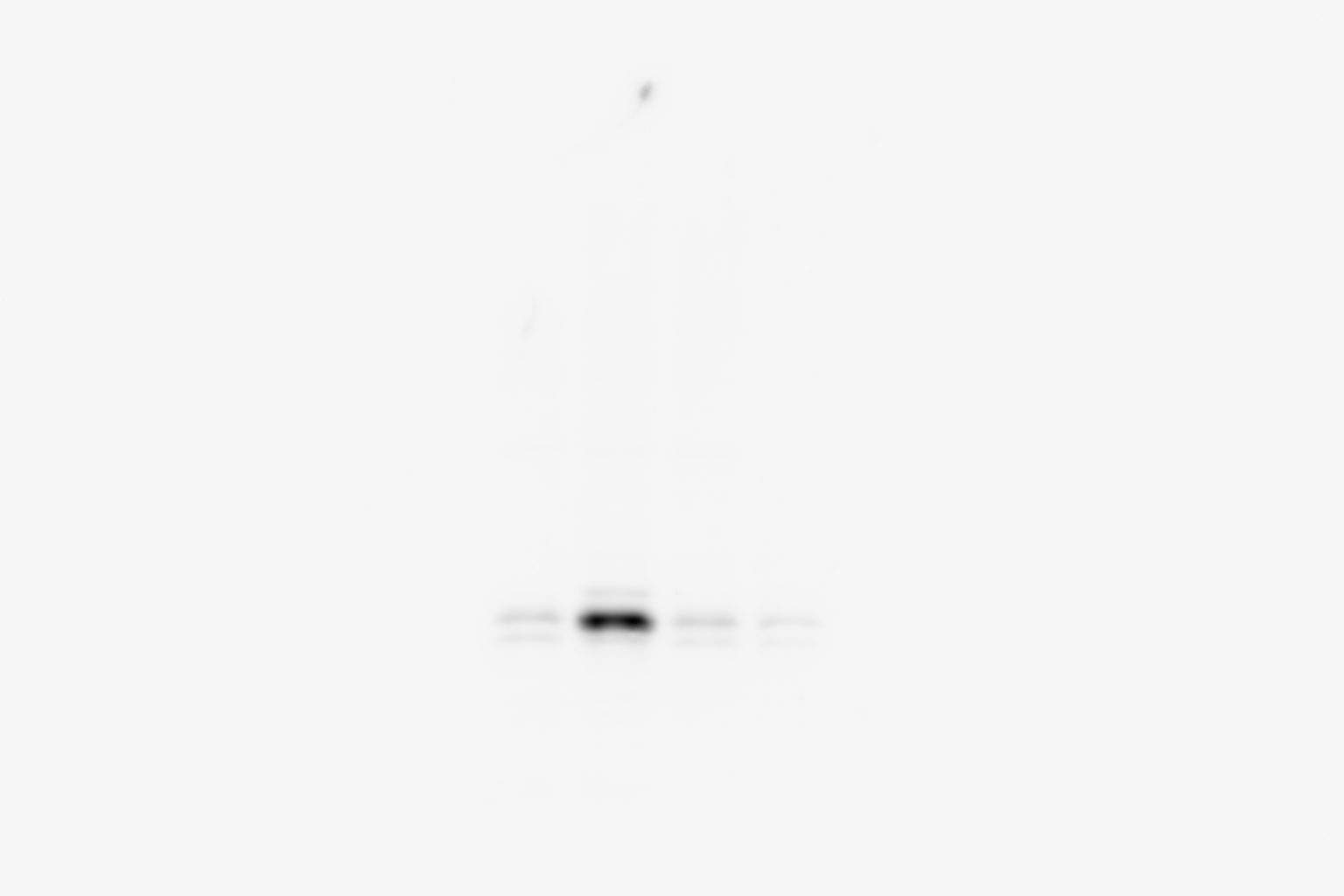

Supplement: Figure 1—source data 1. [file elife-104374-fig1-data1.zip › Figure1-source data 1/Figure 1L Snail.tif]

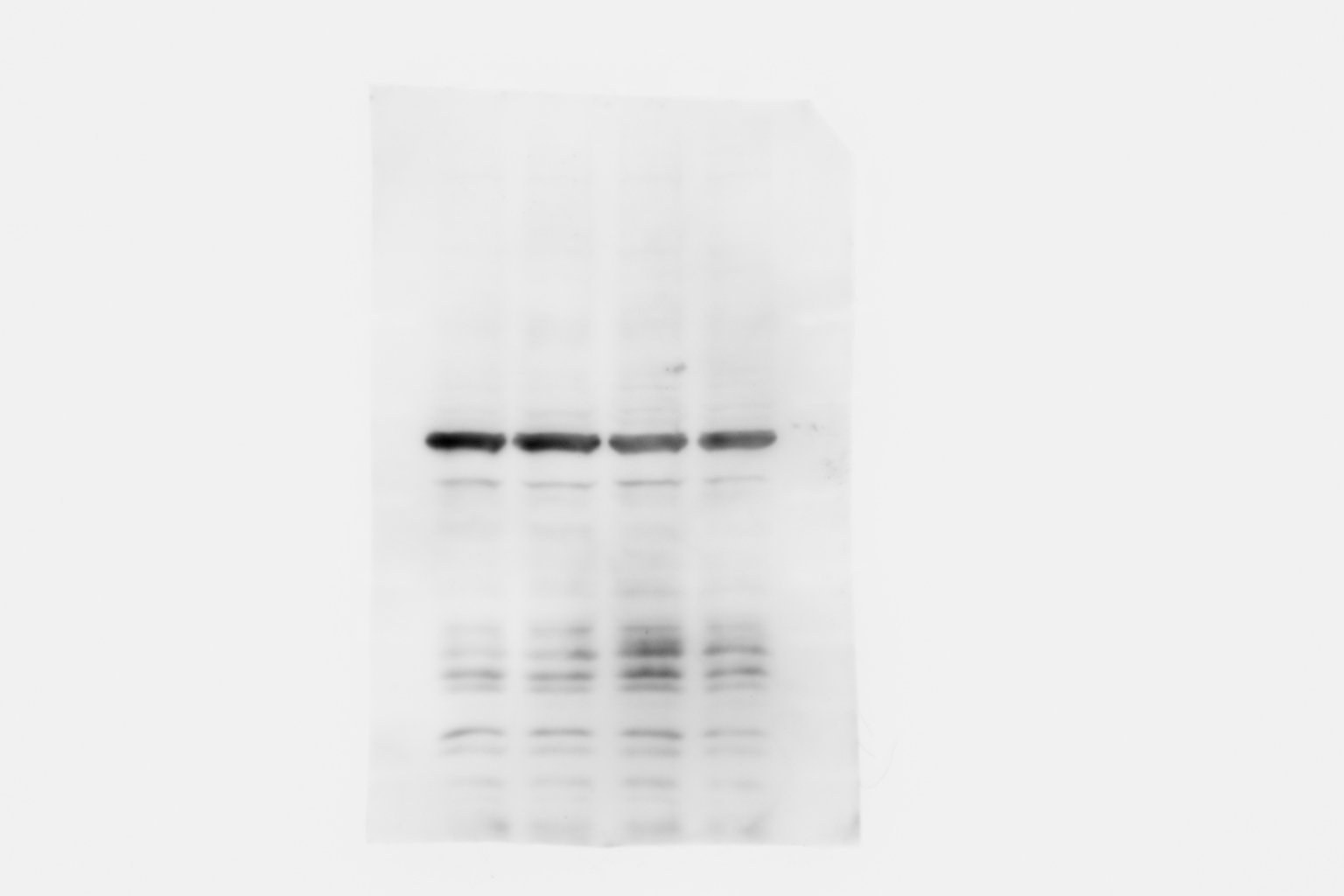

Supplement: Figure 1—source data 1. [file elife-104374-fig1-data1.zip › Figure1-source data 1/Figure 1L tubulin.tif]

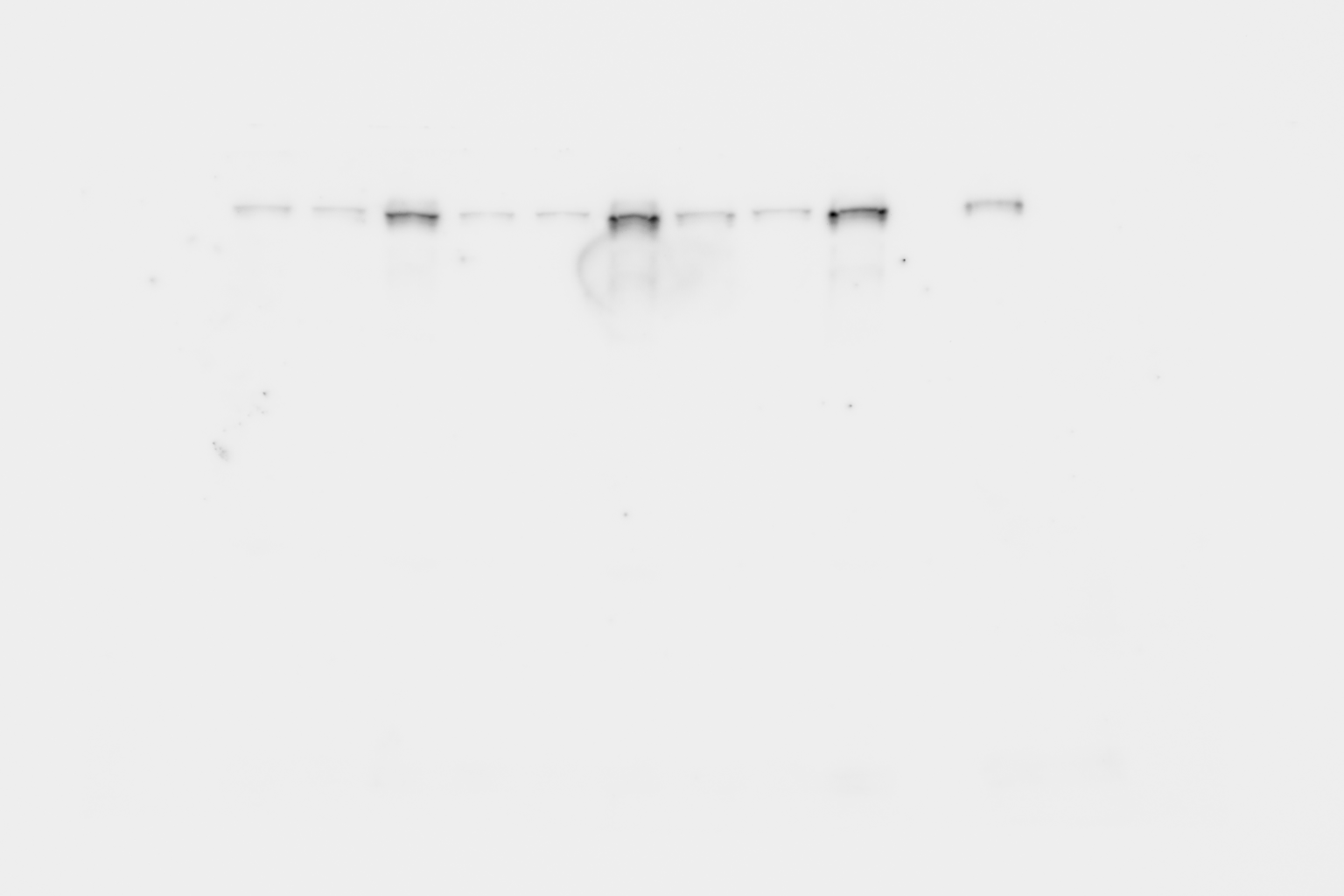

Supplement: Figure 1—source data 1. [file elife-104374-fig1-data1.zip › Figure1-source data 1/Figure 1N ABCA1.tif]

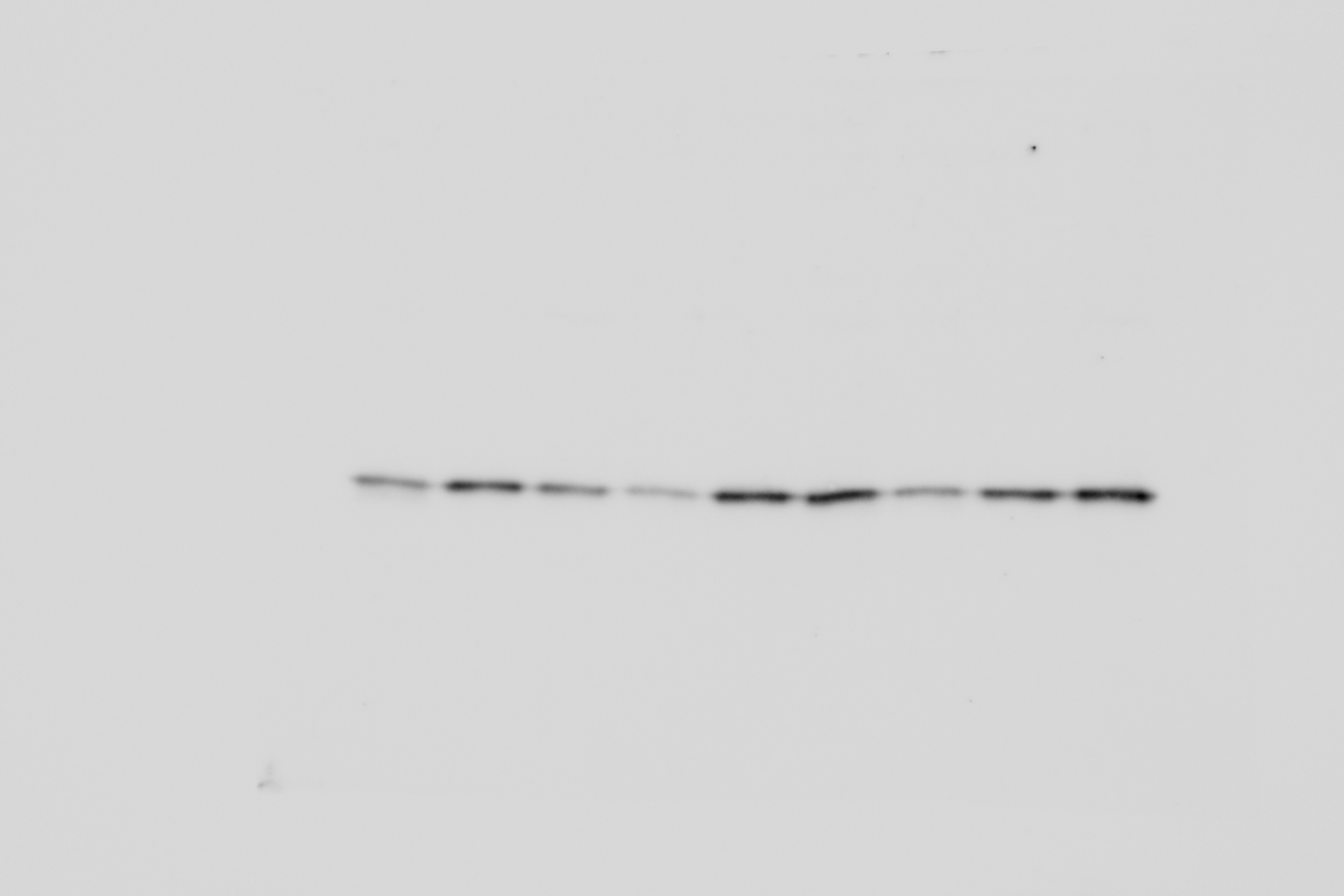

Supplement: Figure 1—source data 1. [file elife-104374-fig1-data1.zip › Figure1-source data 1/Figure 1N Snail.tif]

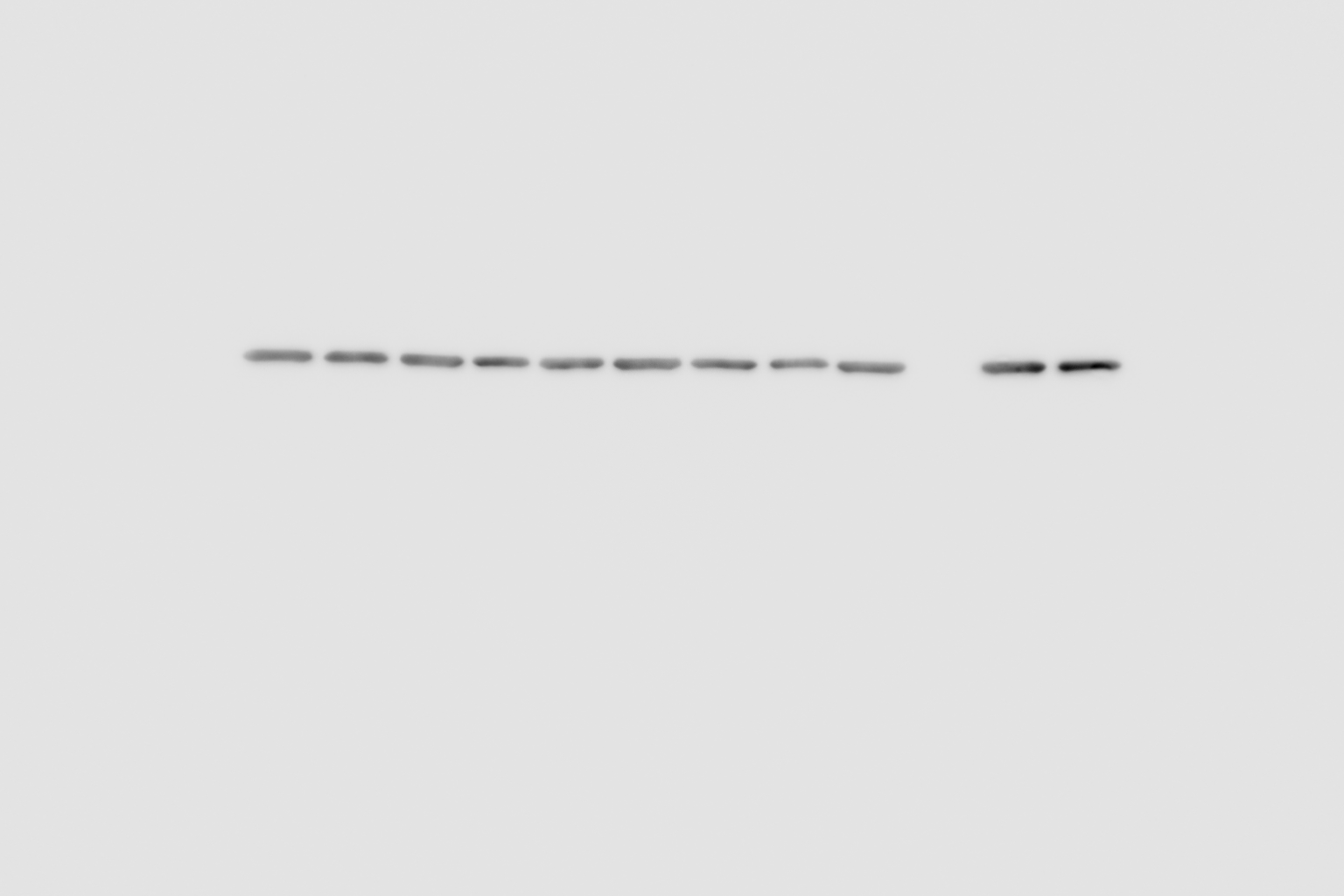

Supplement: Figure 1—source data 1. [file elife-104374-fig1-data1.zip › Figure1-source data 1/Figure 1N tubulin.tif]

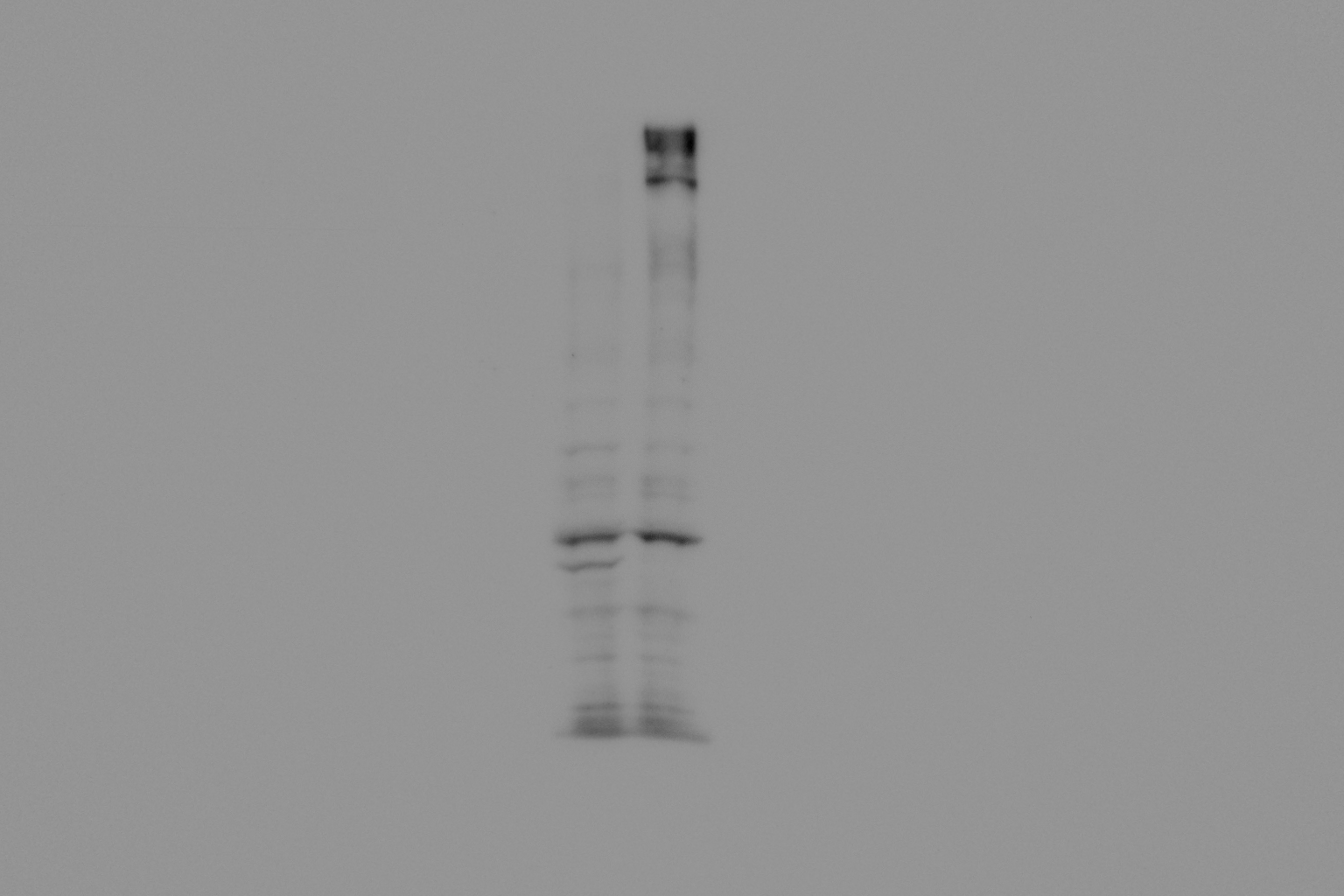

Supplement: Figure 1—source data 1. [file elife-104374-fig1-data1.zip › Figure1-source data 1/Figure 1O ABCA1.tif]

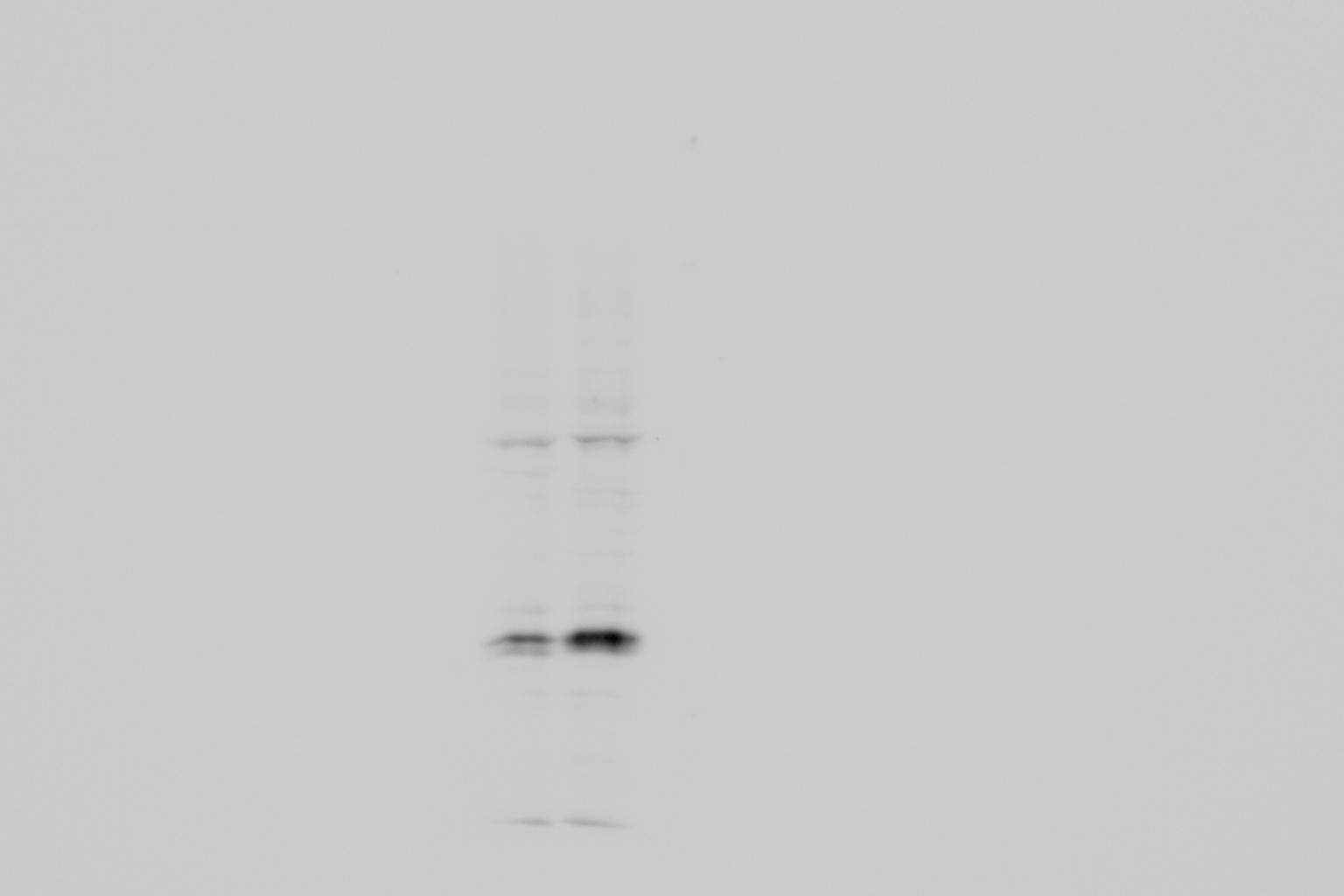

Supplement: Figure 1—source data 1. [file elife-104374-fig1-data1.zip › Figure1-source data 1/Figure 1O Snail.tif]

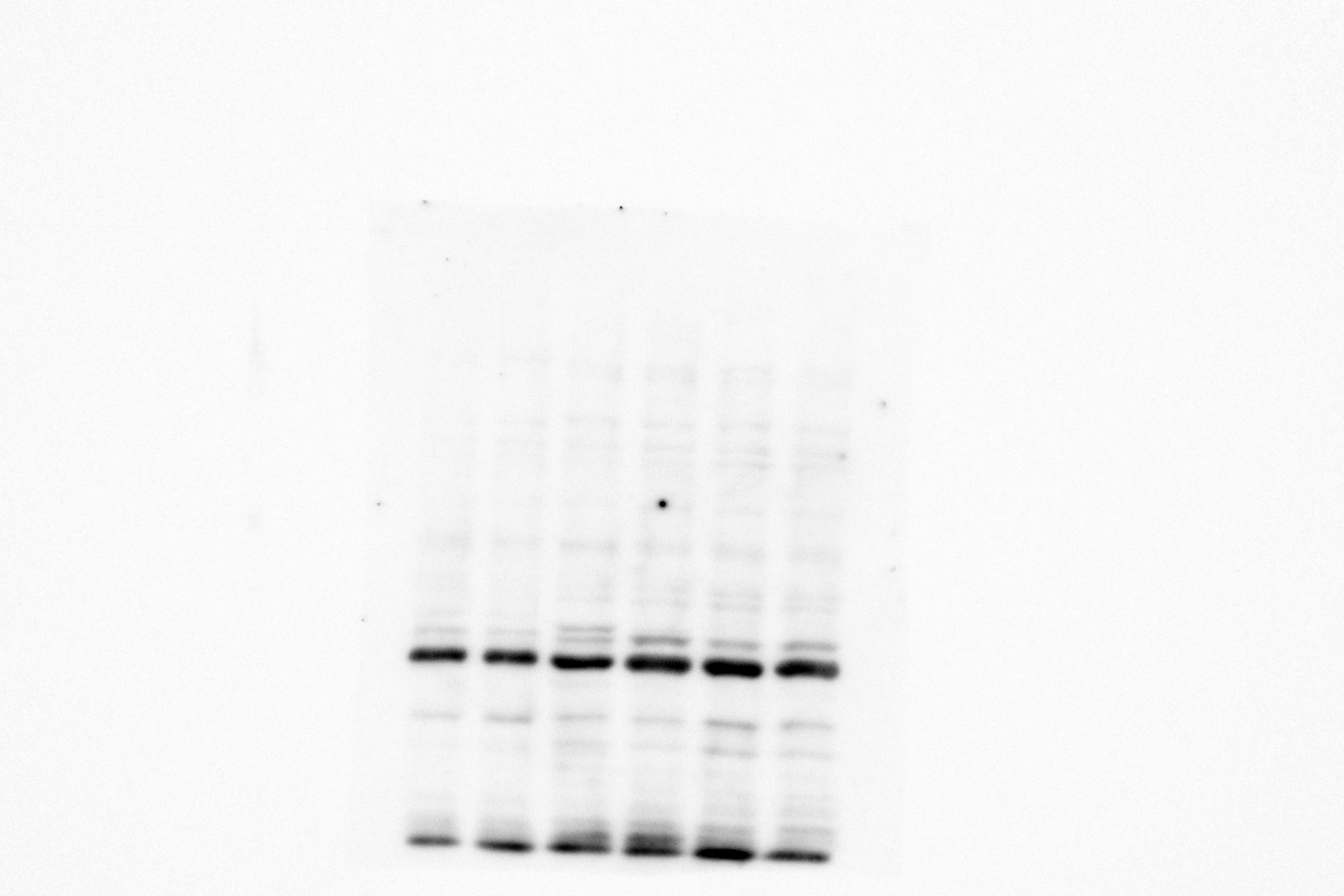

Supplement: Figure 1—source data 1. [file elife-104374-fig1-data1.zip › Figure1-source data 1/Figure 1O tubulin.tif]

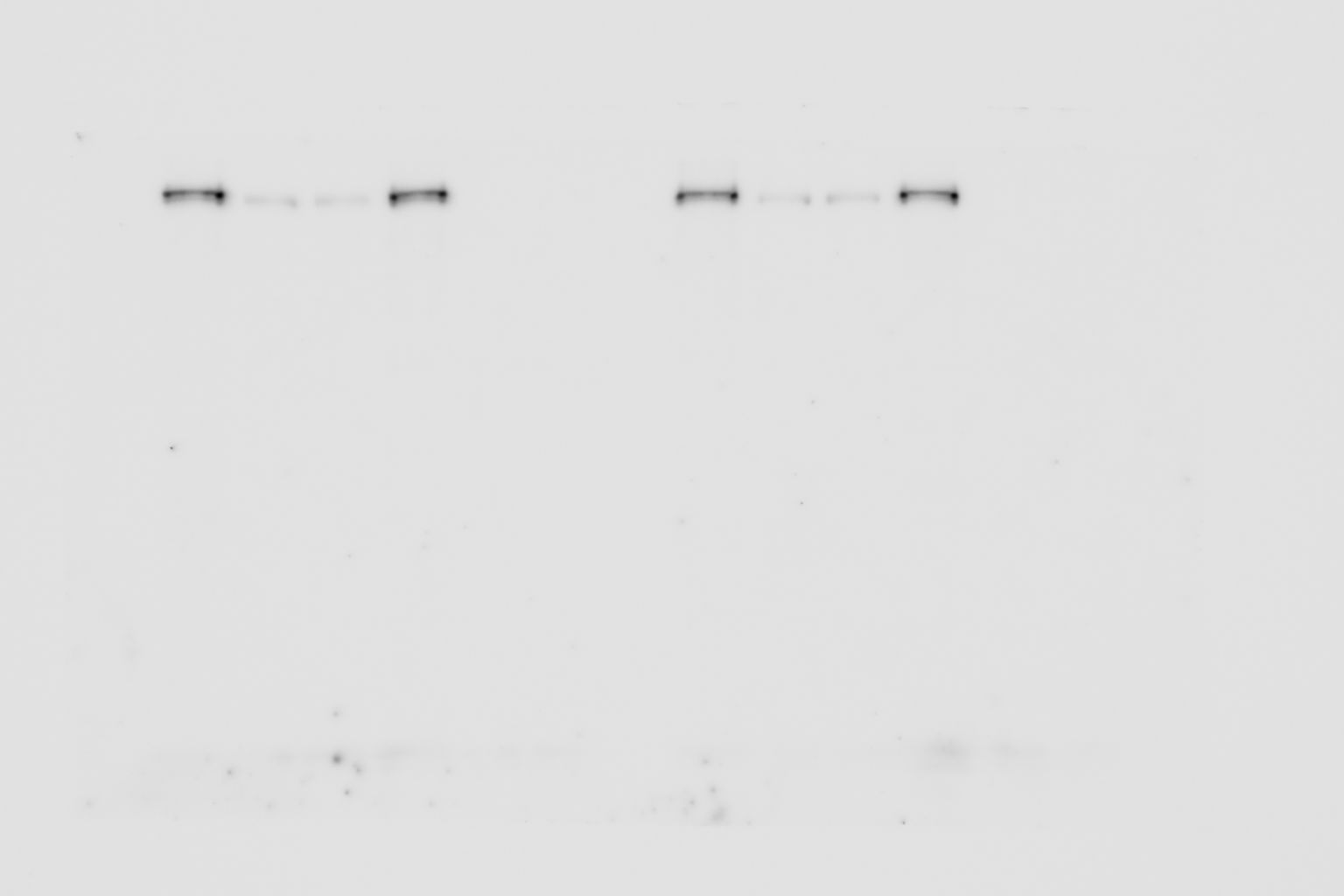

Supplement: Figure 2—source data 1. [file elife-104374-fig2-data1.zip › Figure2-source data 1/Figure 2D ABCA1.tif]

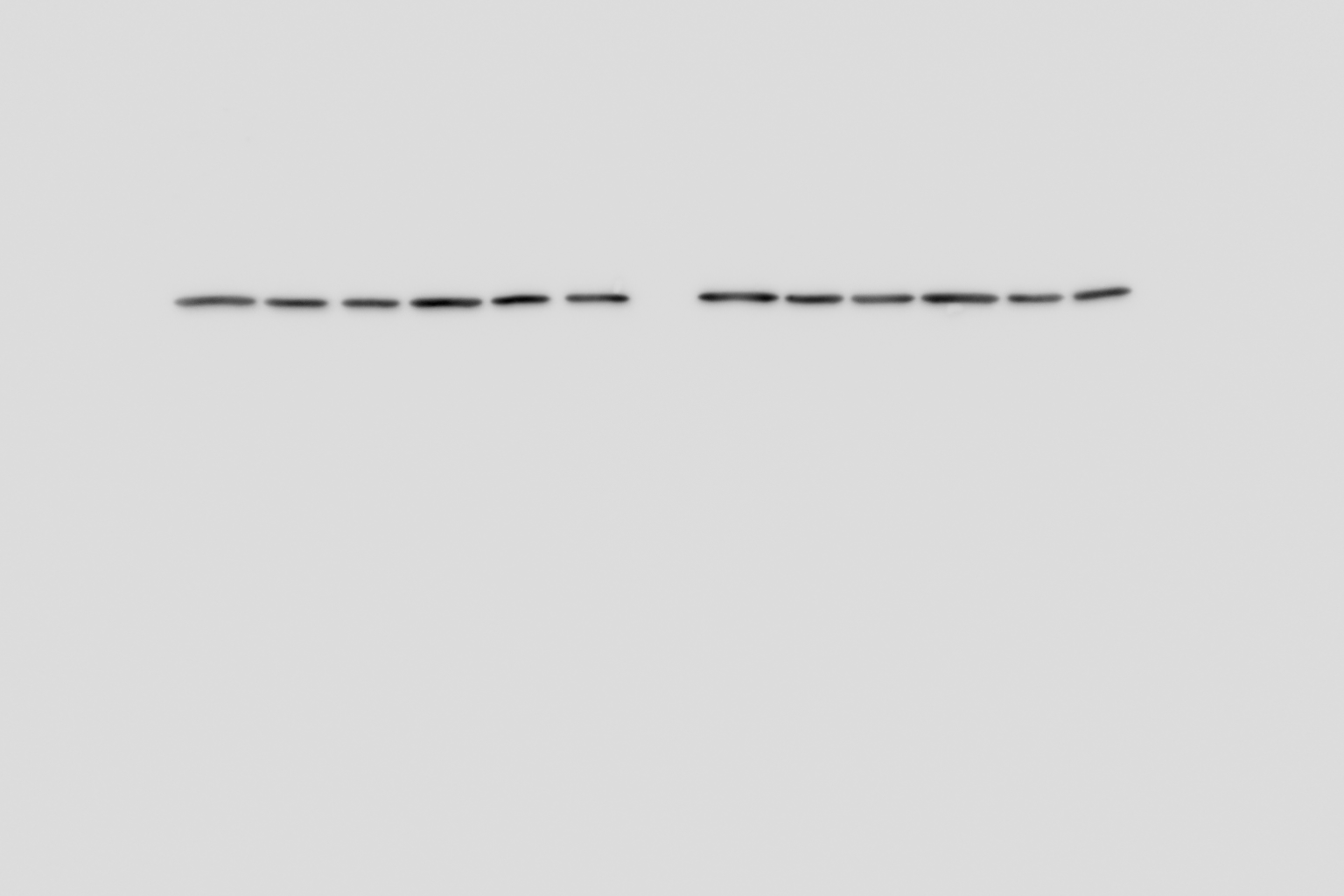

Supplement: Figure 2—source data 1. [file elife-104374-fig2-data1.zip › Figure2-source data 1/Figure 2D tubulin.tif]

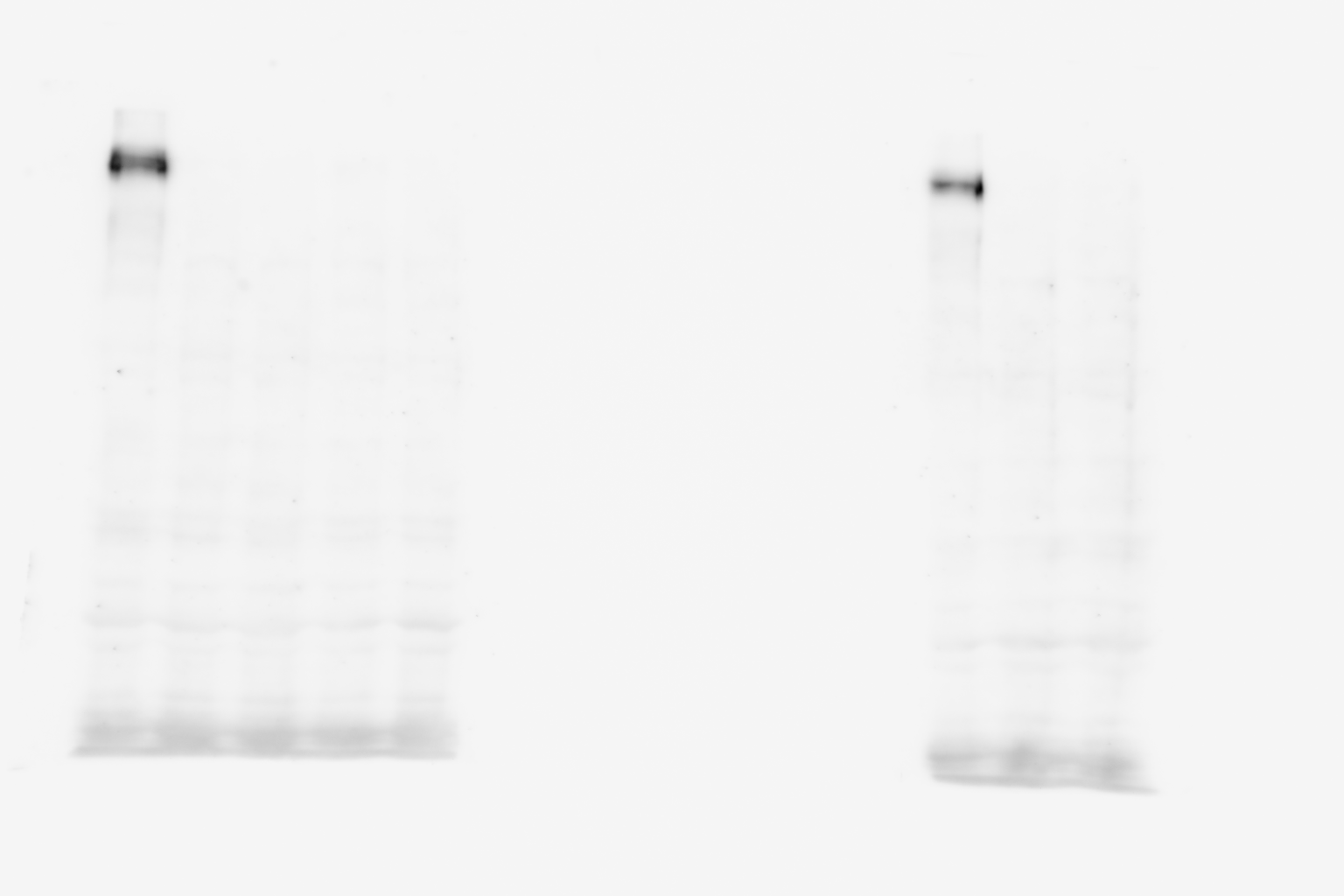

Supplement: Figure 2—source data 1. [file elife-104374-fig2-data1.zip › Figure2-source data 1/Figure2-figure_supplement1A_ABCA1.tif]

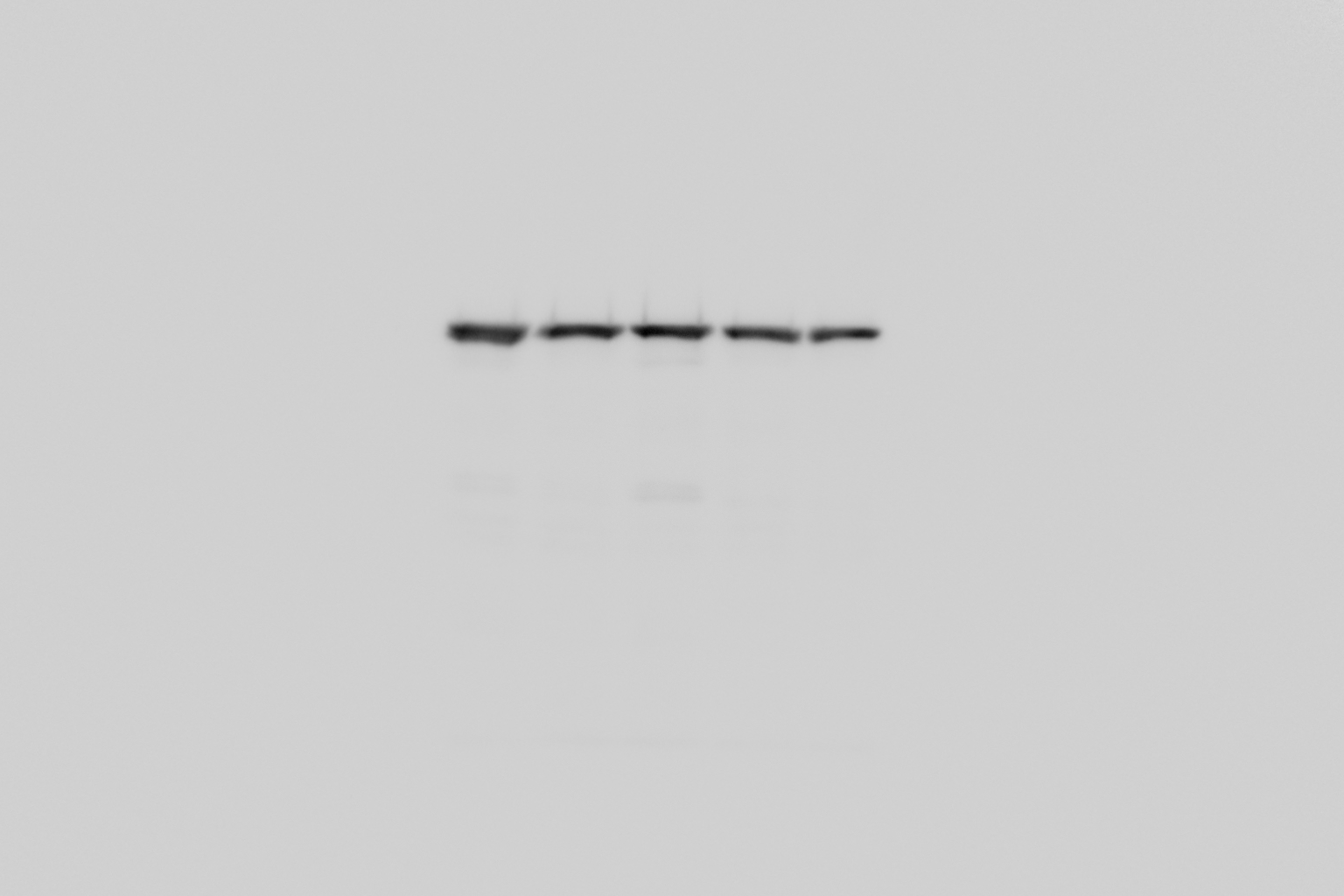

Supplement: Figure 2—source data 1. [file elife-104374-fig2-data1.zip › Figure2-source data 1/Figure2-figure_supplement1A_Akt.tif]

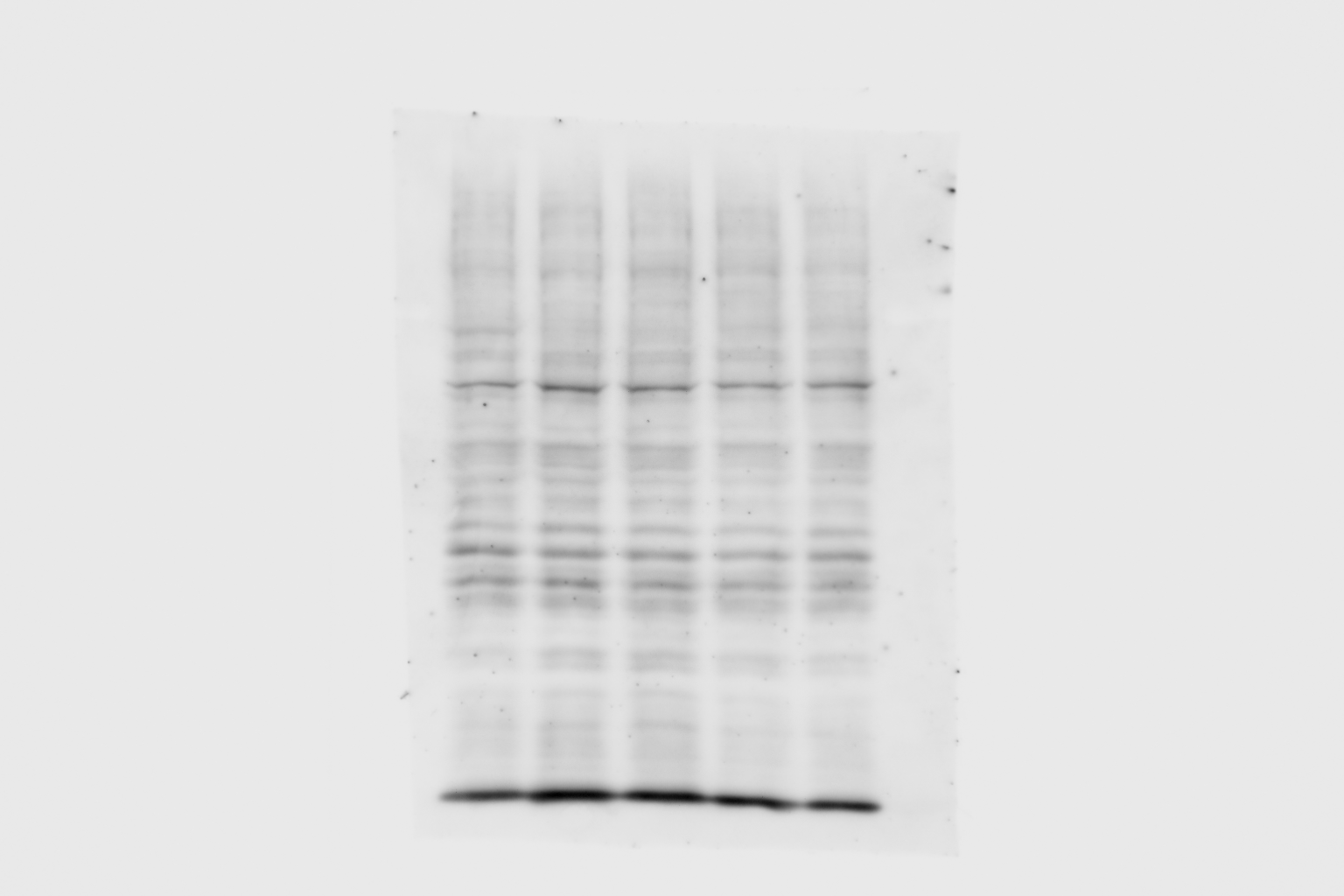

Supplement: Figure 2—source data 1. [file elife-104374-fig2-data1.zip › Figure2-source data 1/Figure2-figure_supplement1A_p-Akt.tif]

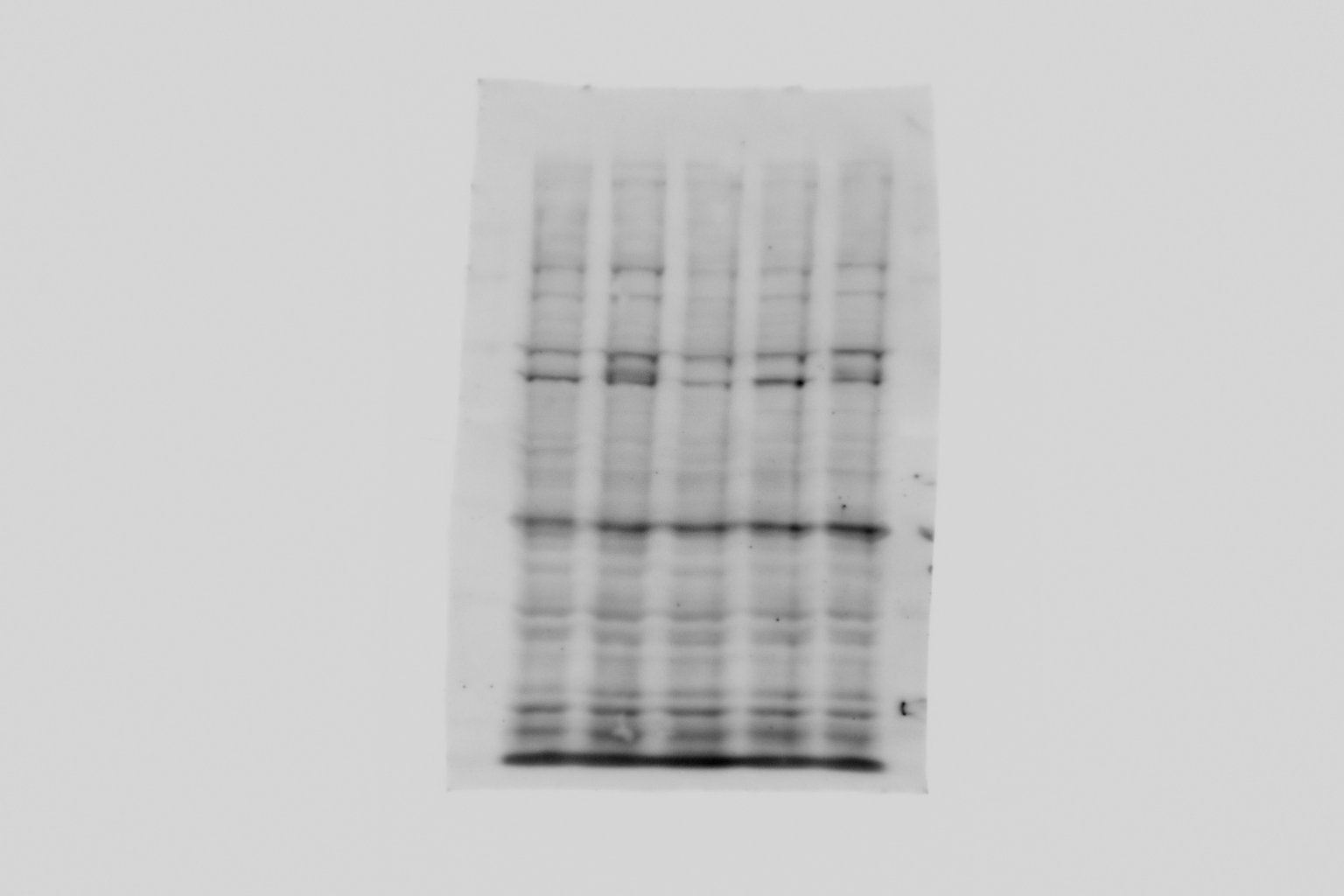

Supplement: Figure 2—source data 1. [file elife-104374-fig2-data1.zip › Figure2-source data 1/Figure2-figure_supplement1A_pFoxO3a.tif]

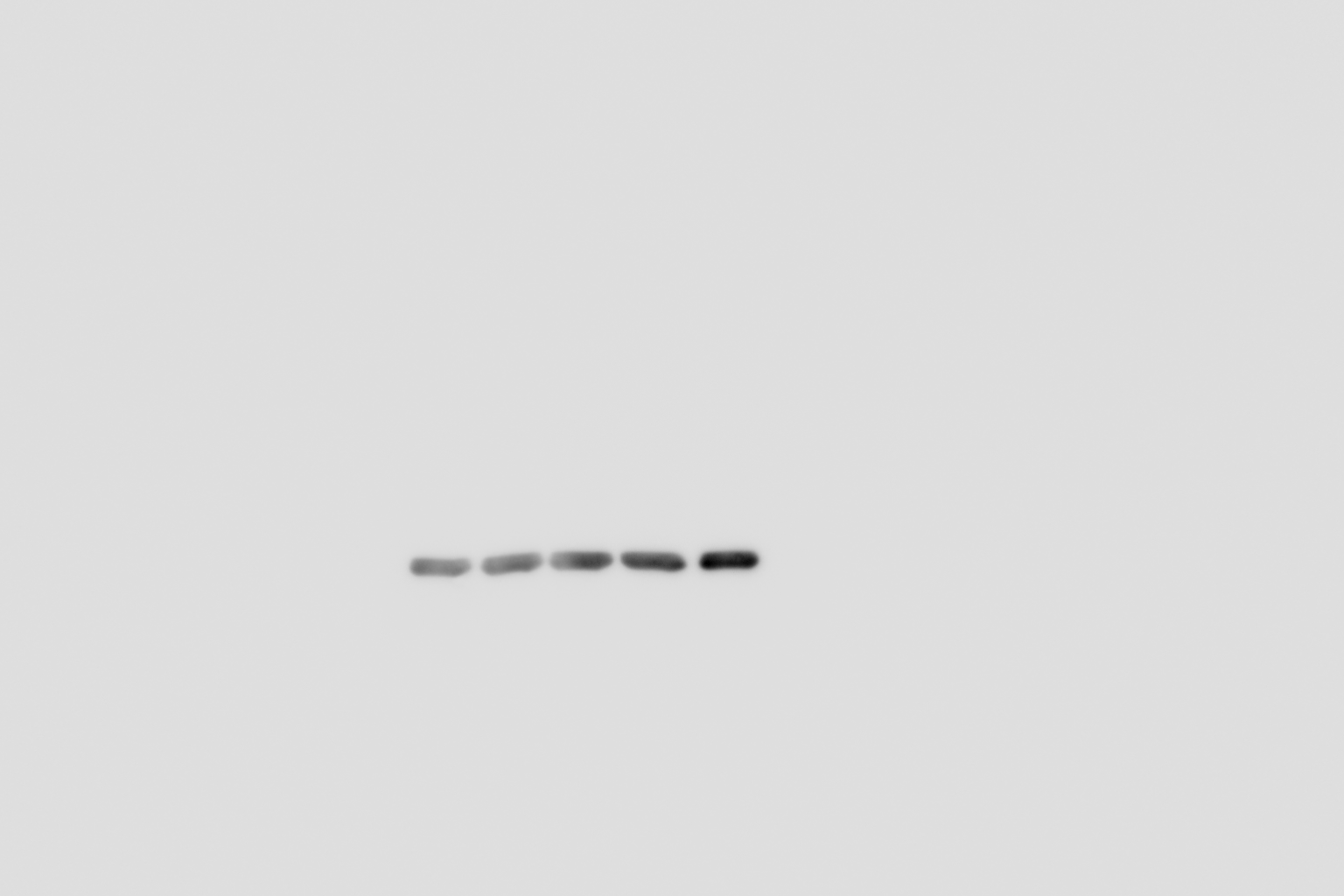

Supplement: Figure 2—source data 1. [file elife-104374-fig2-data1.zip › Figure2-source data 1/Figure2-figure_supplement1A_tubulin.tif]

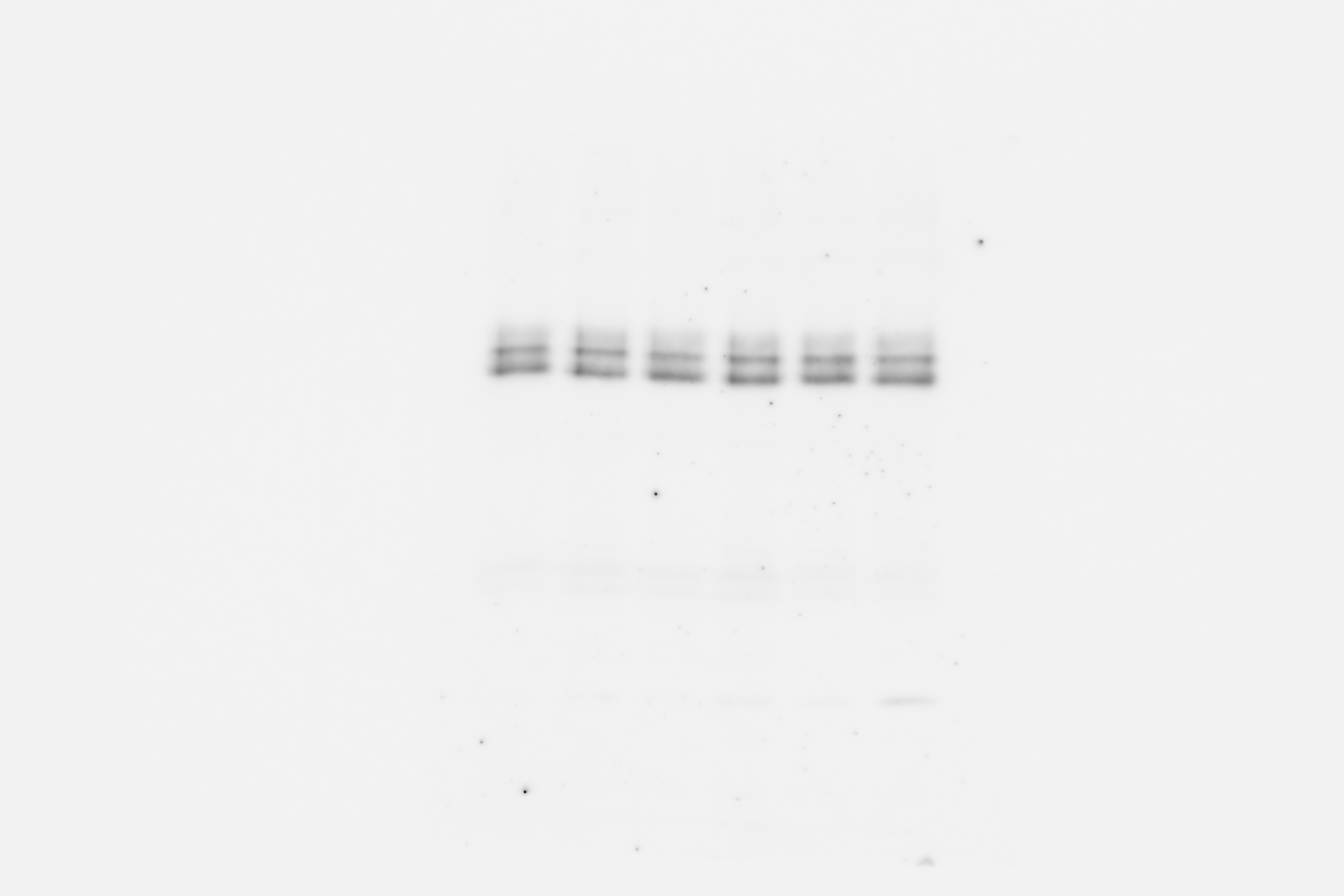

Supplement: Figure 2—source data 1. [file elife-104374-fig2-data1.zip › Figure2-source data 1/Figure2-figure_supplement1B_c-myc.tif]

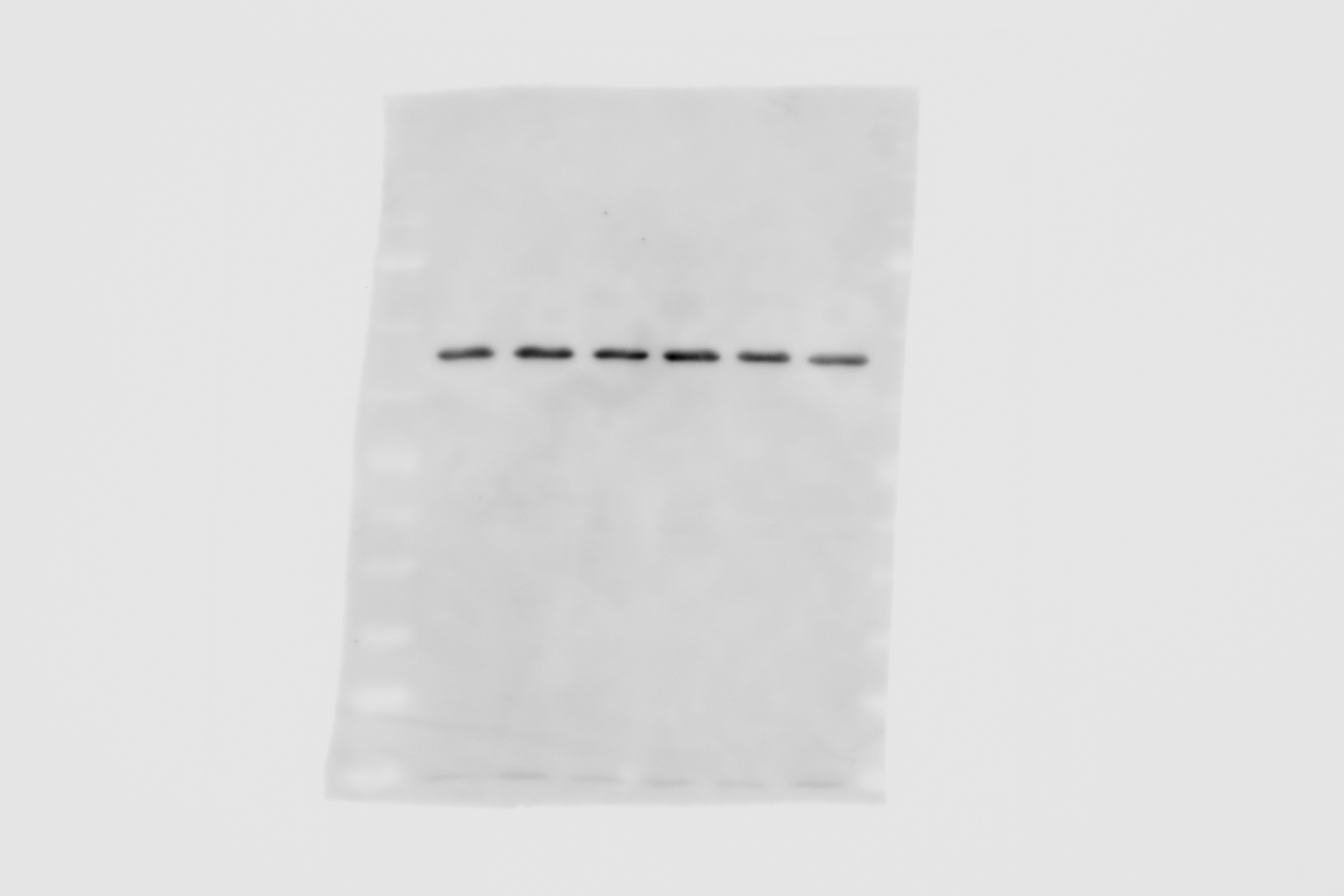

Supplement: Figure 2—source data 1. [file elife-104374-fig2-data1.zip › Figure2-source data 1/Figure2-figure_supplement1B_tubulin.tif]

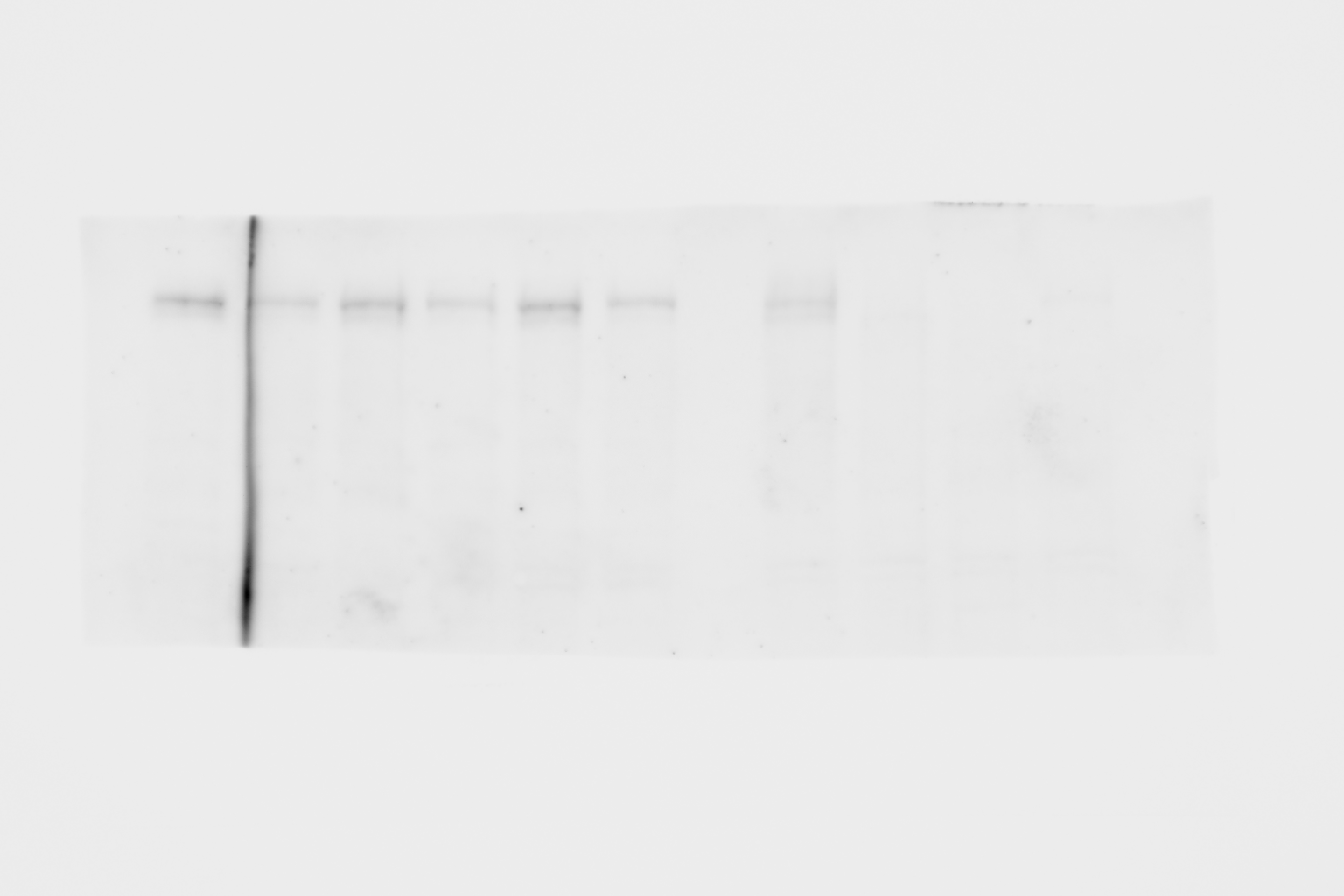

Supplement: Figure 2—source data 1. [file elife-104374-fig2-data1.zip › Figure2-source data 1/Figure2-figure_supplement2A_ABCA1.tif]

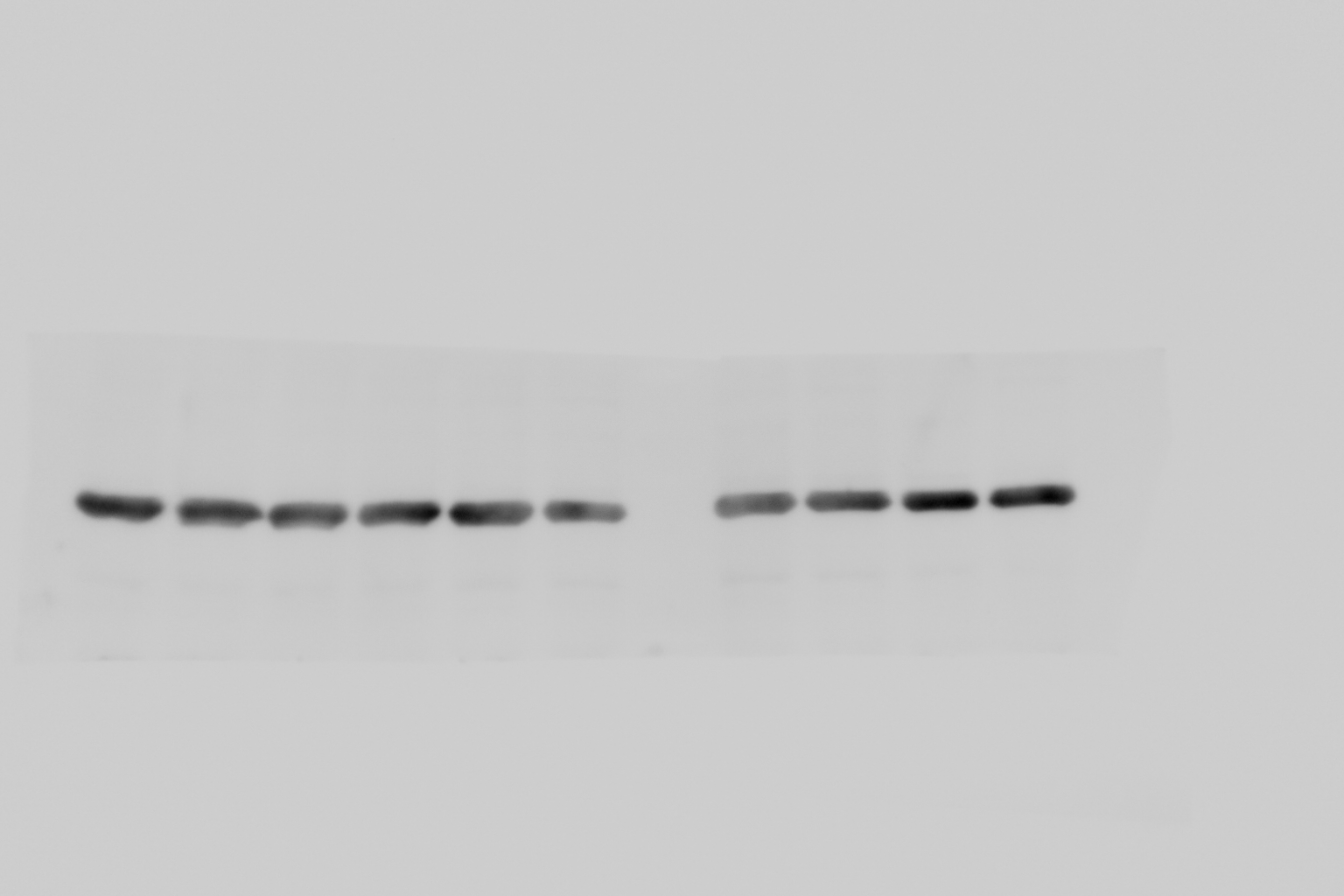

Supplement: Figure 2—source data 1. [file elife-104374-fig2-data1.zip › Figure2-source data 1/Figure2-figure_supplement2A_tubulin.tif]

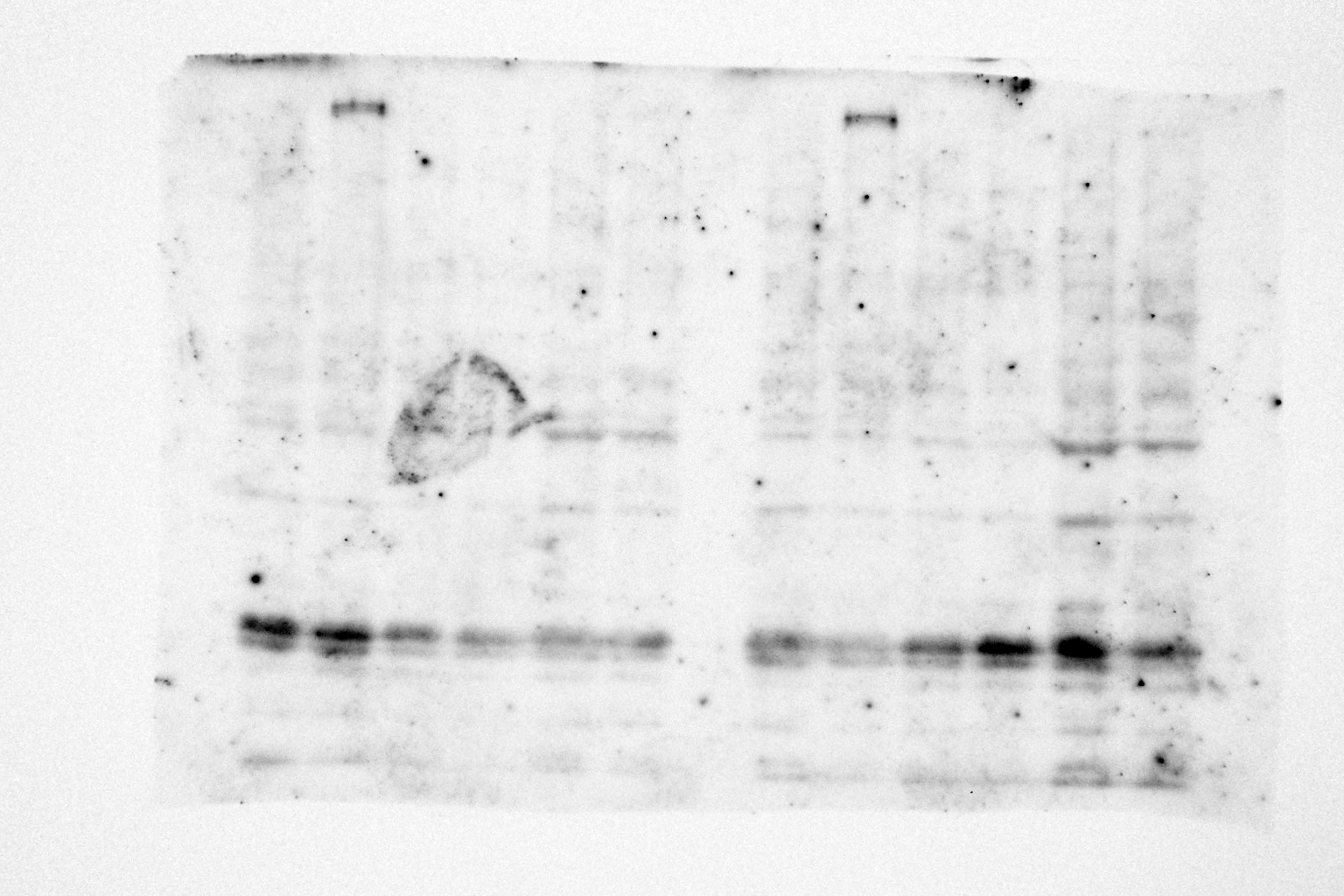

Supplement: Figure 3—source data 1. [file elife-104374-fig3-data1.zip › Figure3-source data 1/Figure 3I ABCA1.tif]

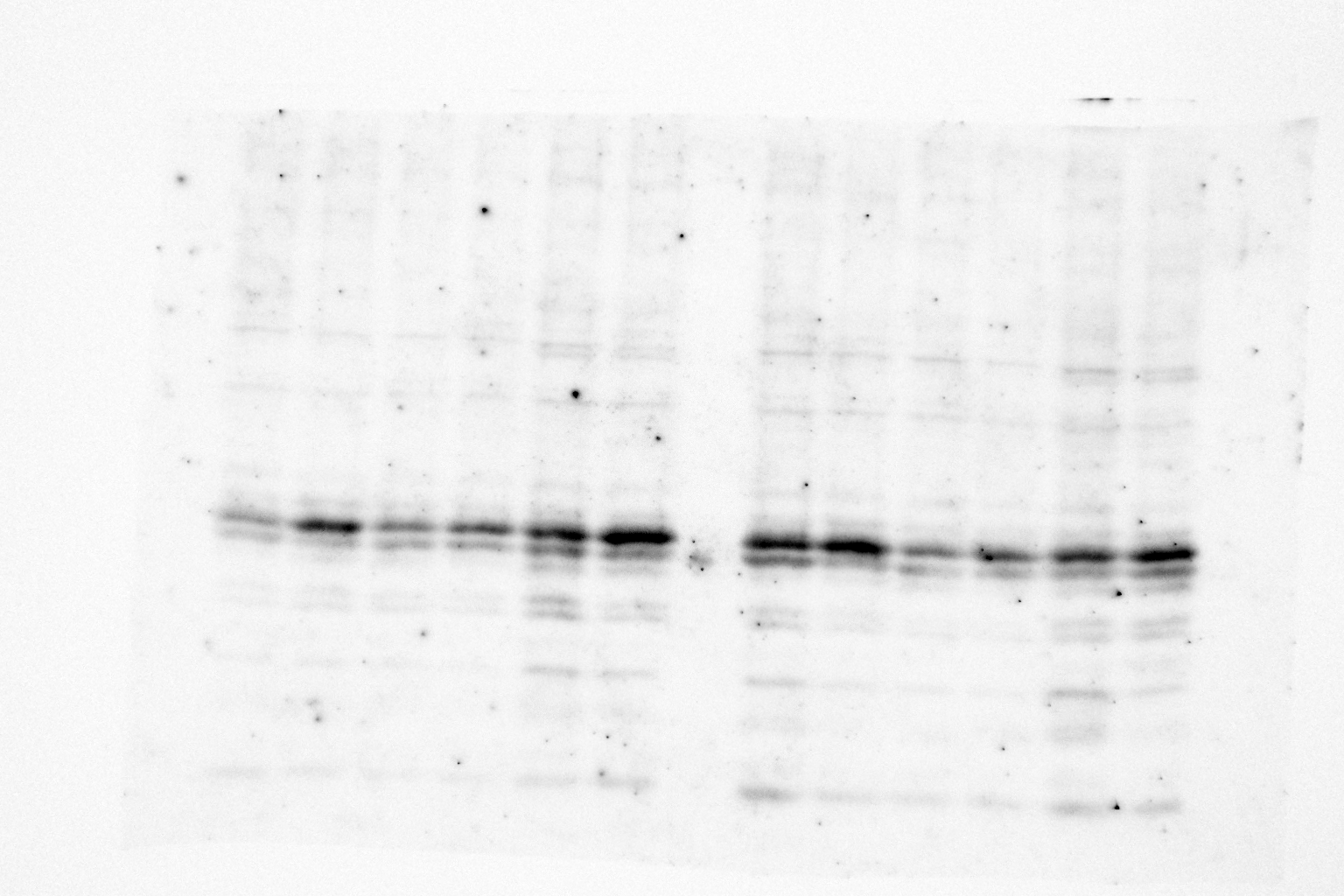

Supplement: Figure 3—source data 1. [file elife-104374-fig3-data1.zip › Figure3-source data 1/Figure 3I Snail.tif]

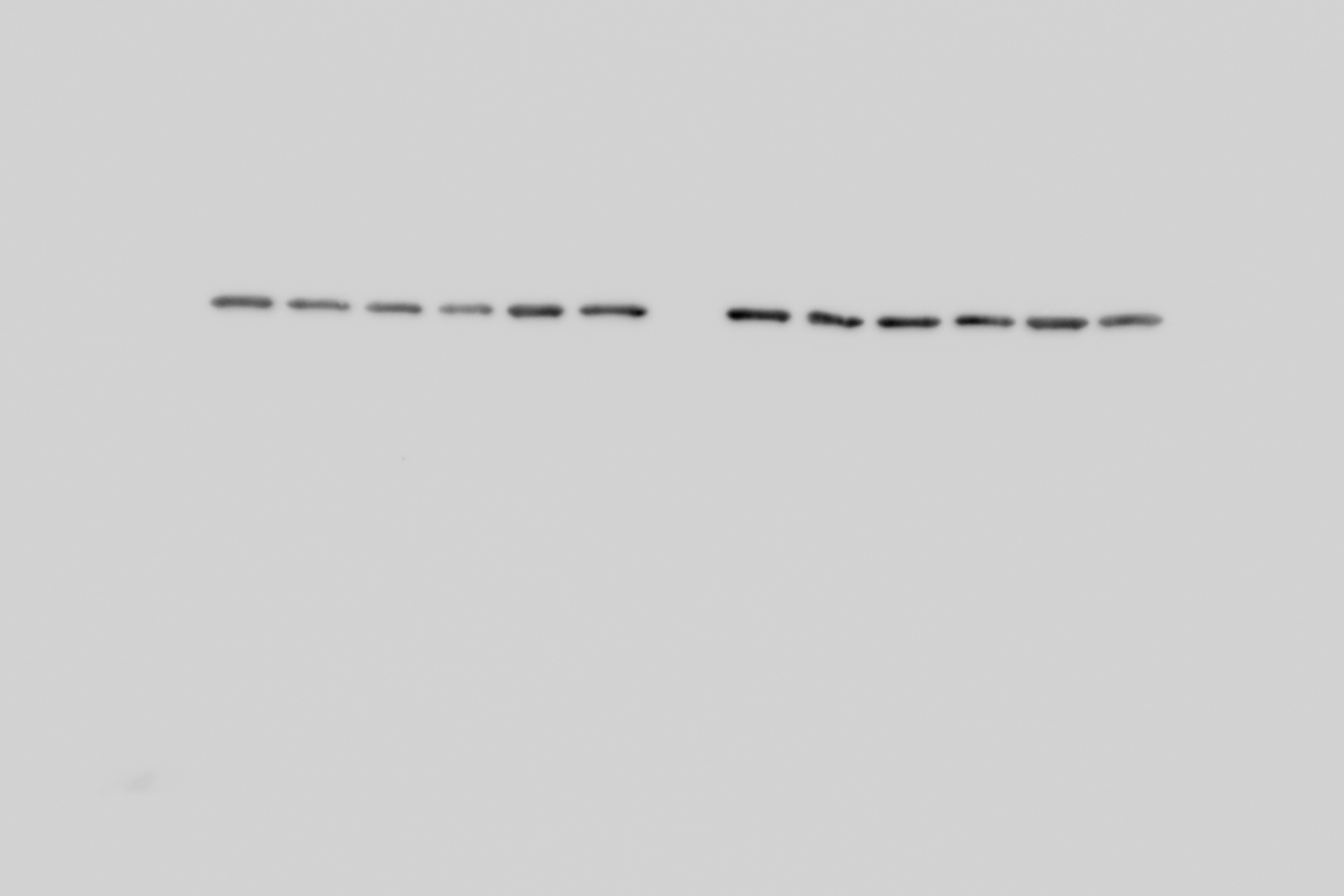

Supplement: Figure 3—source data 1. [file elife-104374-fig3-data1.zip › Figure3-source data 1/Figure 3I tubulin.tif]

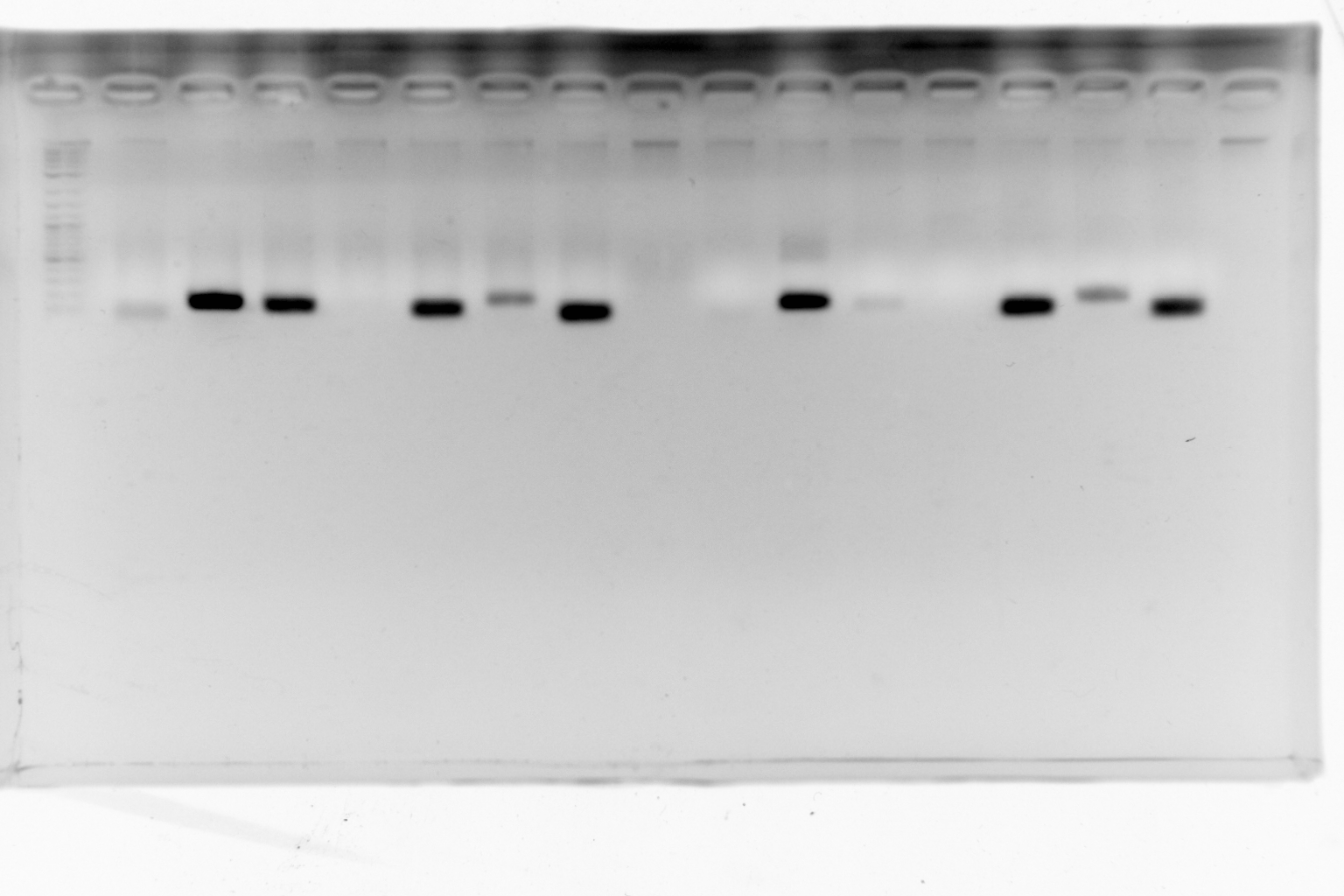

Supplement: Figure 3—source data 1. [file elife-104374-fig3-data1.zip › Figure3-source data 1/Figure3F CERS.tiff]

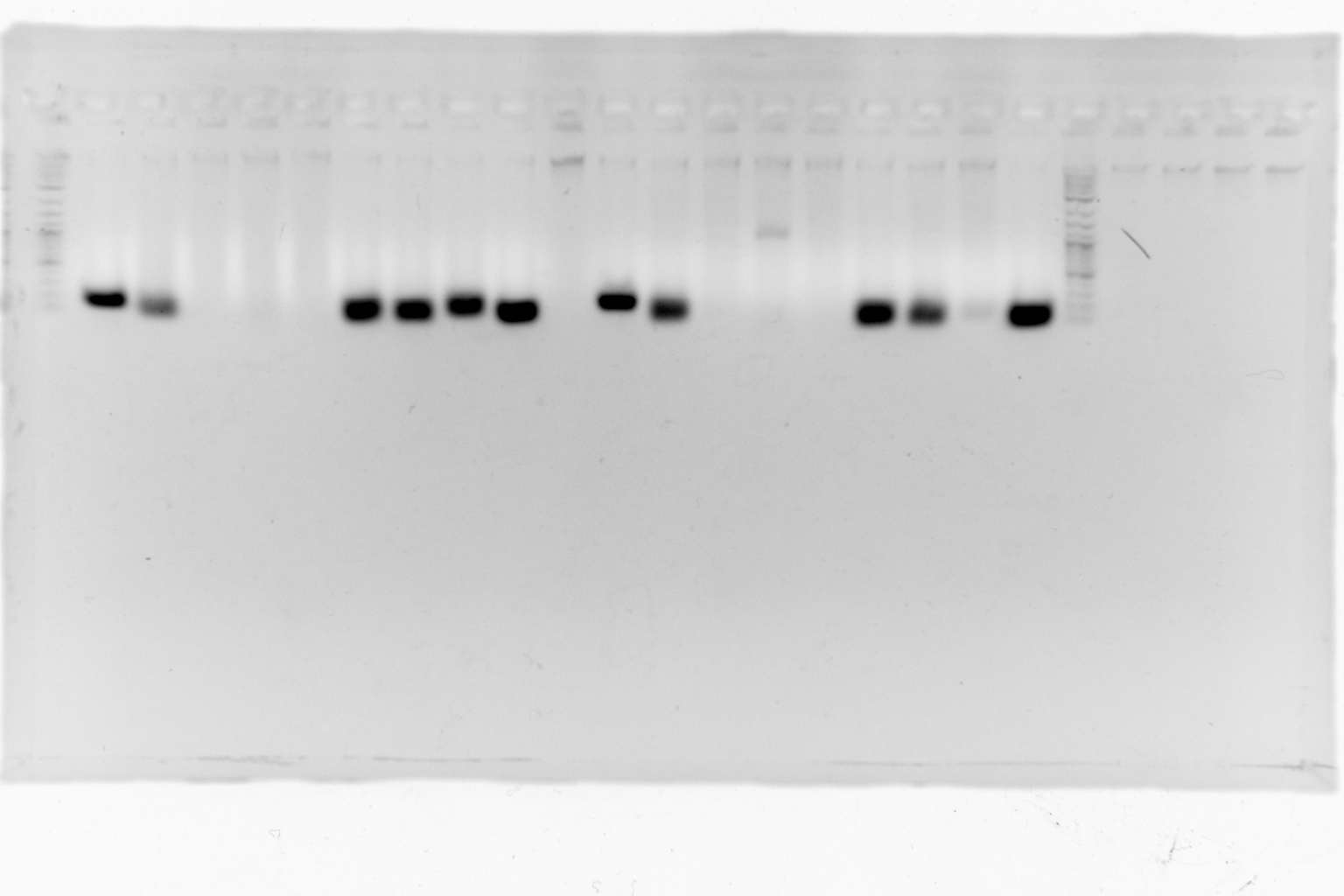

Supplement: Figure 3—source data 1. [file elife-104374-fig3-data1.zip › Figure3-source data 1/Figure3F ELOVL.tiff]

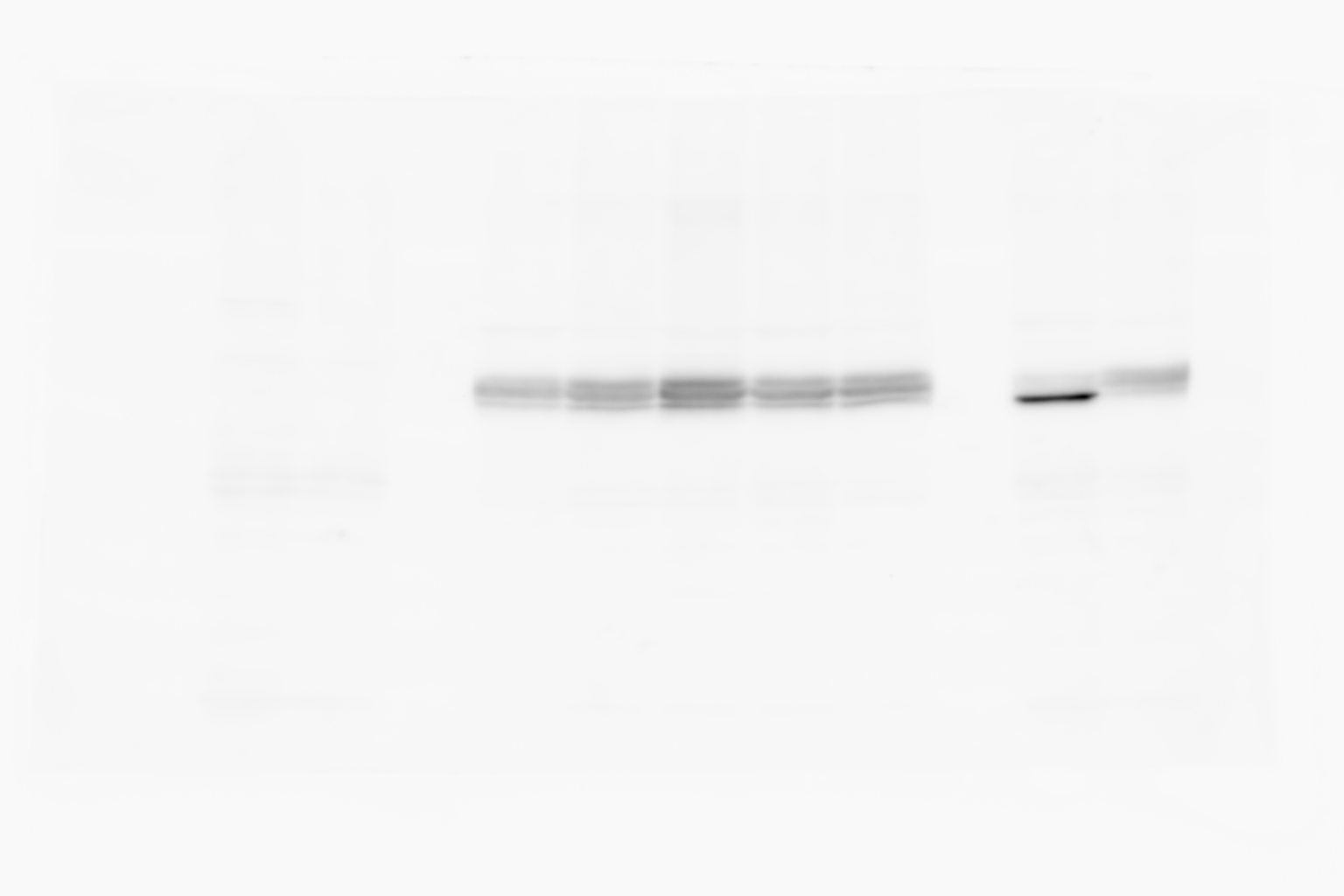

Supplement: Figure 4—source data 1. [file elife-104374-fig4-data1.zip › Figure4-source data 1/Figure 4B SOAT1.tif]

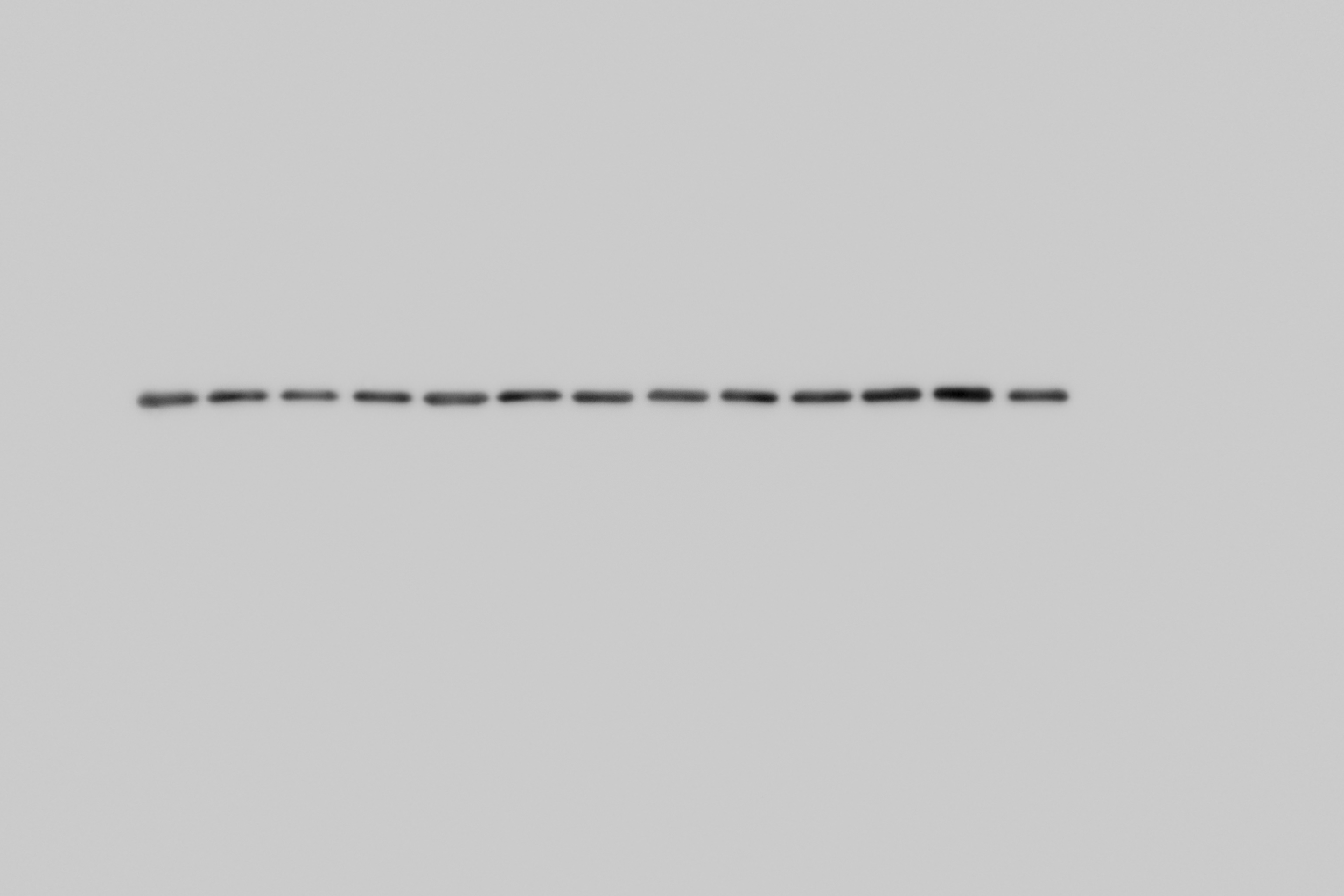

Supplement: Figure 4—source data 1. [file elife-104374-fig4-data1.zip › Figure4-source data 1/Figure 4B tubulin.tif]

Figure 4B SOAT1

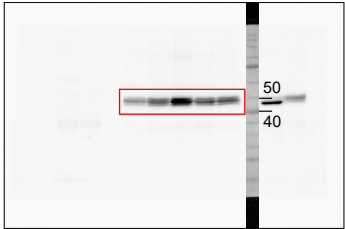

Figure 4B  $\alpha$ -tubulin

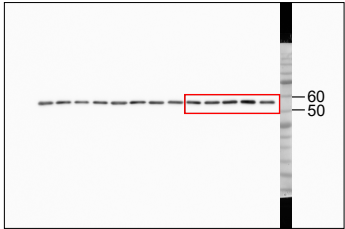

Supplement: Figure 4—source data 2. [file elife-104374-fig4-data2.pdf]
