# Supplementary material for: Chemotherapy resistance due to epithelial-to-mesenchymal transition is caused by abnormal lipid metabolic balance
Source: eLife. 2026 Jan 12;13:RP104374. doi: 10.7554/eLife.104374 (PMC12795503; doi:10.7554/eLife.104374)

Figure 1B ABCA1

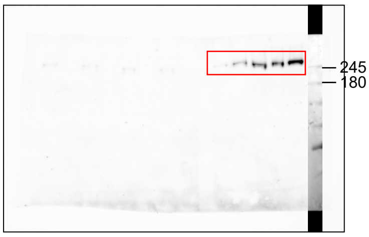

Figure 1B Snail

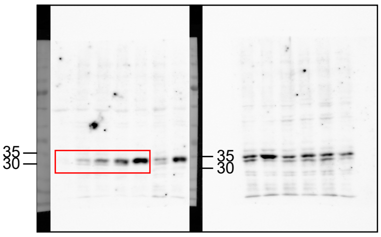

Figure 1B α-Tubulin

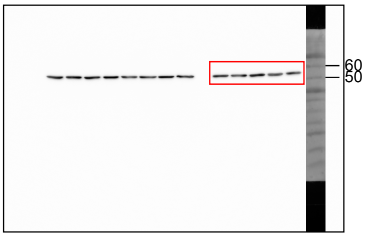

Figure 1L ABCA1

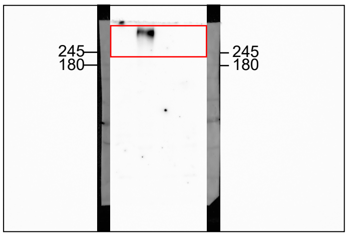

Figure 1L Snail

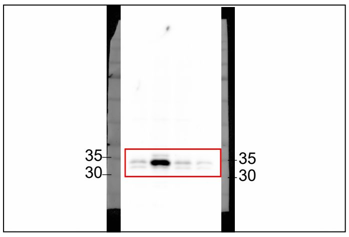

Figure 1L E-cadherin

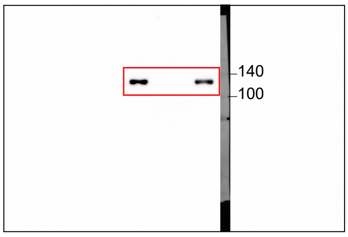

Figure 1L α-Tubulin

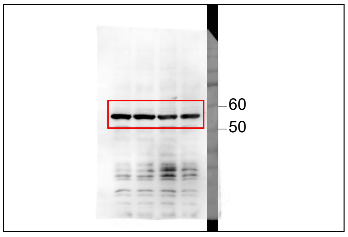

Figure 1N ABCA1

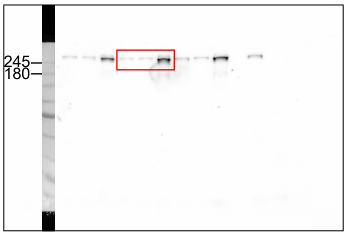

Figure 1N Snail

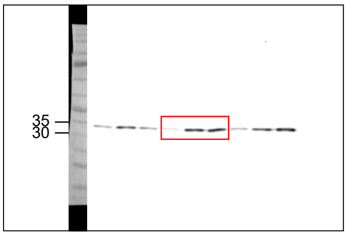

Figure 1N α-tubulin

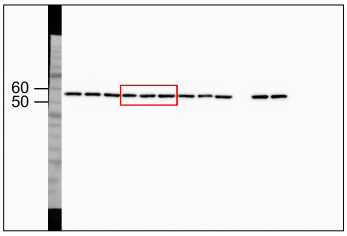

Figure 1O ABCA1

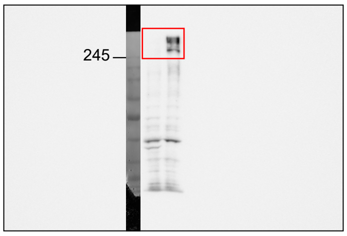

Figure 1O Snail

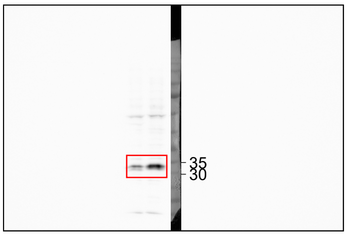

Figure 1O α-tubulin

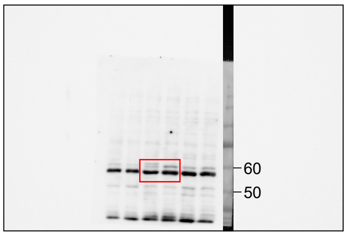

Supplement: Figure 1—source data 2. [file elife-104374-fig1-data2.pdf]
