# Supplementary material for: Chemotherapy resistance due to epithelial-to-mesenchymal transition is caused by abnormal lipid metabolic balance
Source: eLife. 2026 Jan 12;13:RP104374. doi: 10.7554/eLife.104374 (PMC12795503; doi:10.7554/eLife.104374)

Figure 2B ABCA1

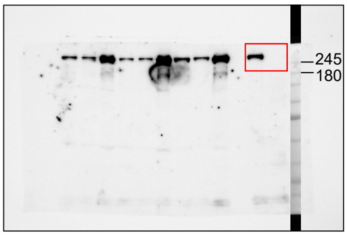

Figure 2B α-tubulin

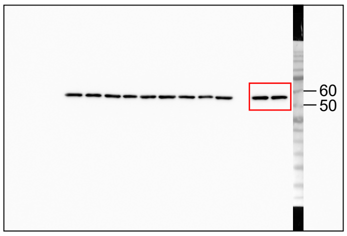

Figure 2D ABCA1

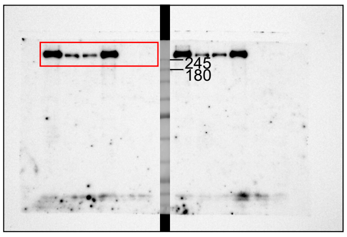

Figure 2D α-tubulin

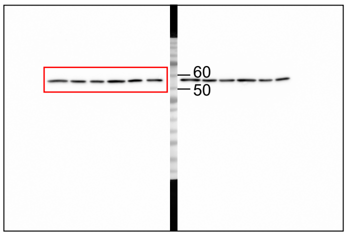

Figure 2 - figure supplement 1A ABCA1

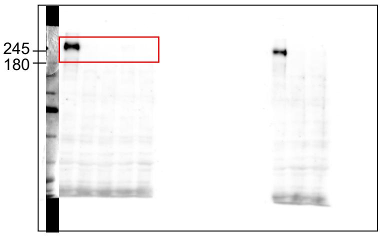

Figure 2 - figure supplement 1A p-FOXO3a

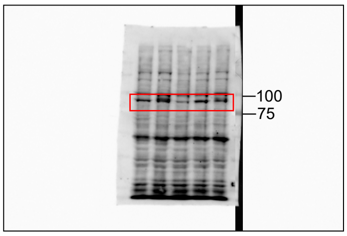

Figure 2 - figure supplement 1A p-Akt

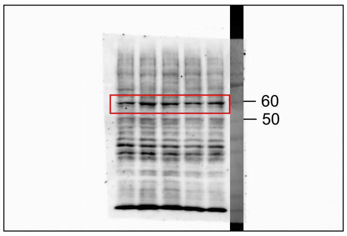

Figure 2 - figure supplement 1A Akt

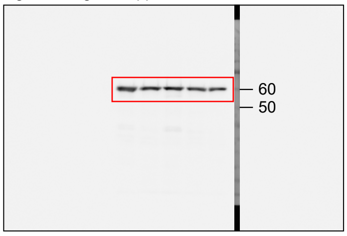

Figure 2 - figure supplement 1A α-tubulin

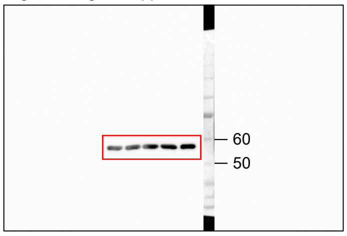

Figure 2 - figure supplement 1B c-myc

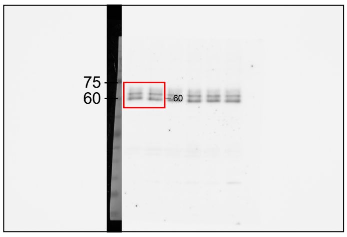

Figure 2 - figure supplement 1B α-tubulin

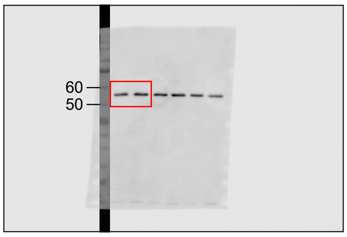

Figure 2 - figure supplement 2A ABCA1

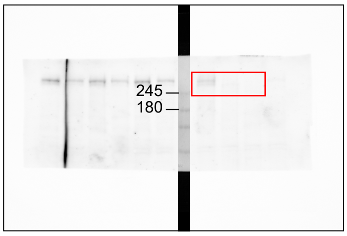

Figure 2 - figure supplement 2A α-tubulin

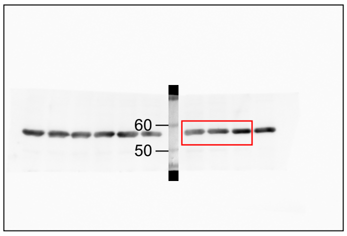

Supplement: Figure 2—source data 2. [file elife-104374-fig2-data2.pdf]
