# Supplementary material for: Chemotherapy resistance due to epithelial-to-mesenchymal transition is caused by abnormal lipid metabolic balance
Source: eLife. 2026 Jan 12;13:RP104374. doi: 10.7554/eLife.104374 (PMC12795503; doi:10.7554/eLife.104374)

Figure 3F *ELOVL*

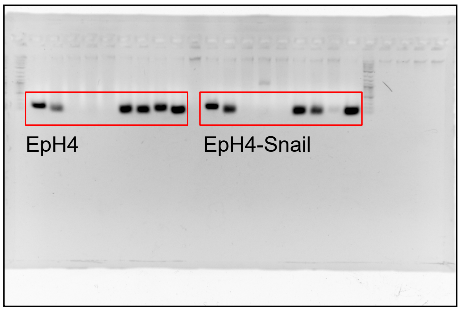

Figure 3F *CERS*

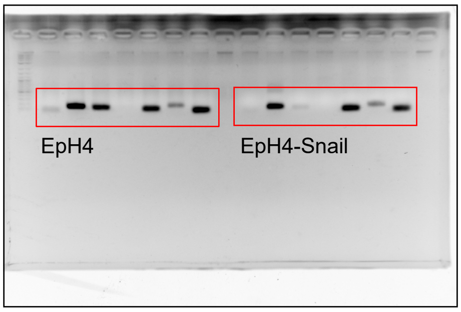

Figure 3H ABCA1

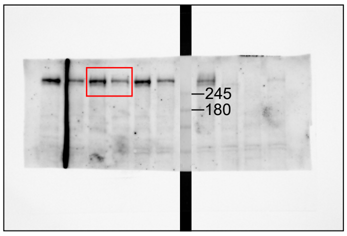

Figure 3H α-tubulin

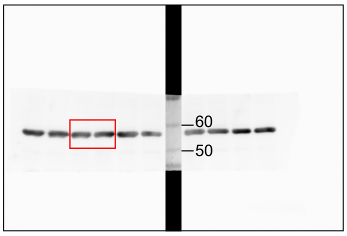

Figure 3I ABCA1

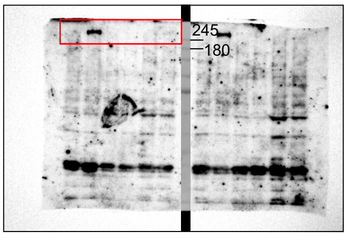

Figure 3I Snail

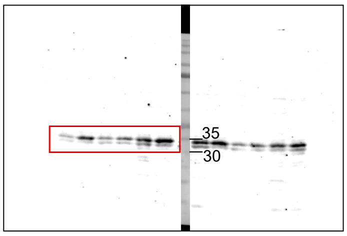

Figure 3I α-tubulin

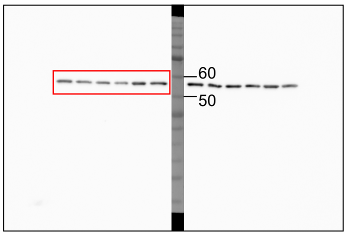

Supplement: Figure 3—source data 2. [file elife-104374-fig3-data2.pdf]
